# Supplementary material for: Trends and influence factors in the prevalence, awareness, treatment, and control of hypertension among US adults from 1999 to 2018
Source: PLoS One. 2023 Sep 28;18(9):e0292159. doi: 10.1371/journal.pone.0292159 (PMC10538741; doi:10.1371/journal.pone.0292159)
Supplement: S1 File — (DOCX) [file pone.0292159.s001.docx]

**Contents of supplement**

[sFigure 1 Flow chart of this study 2](#_Toc132204993)

[sTable 1 Classification of antihypertensive drugs among US hypertension adults from 1999 to 2018 3](#_Toc132204994)

[sTable 2 Definitions of the individual components of the LS7 metric 3](#_Toc132204995)

[sTable 3 Annual prevalence change of hypertension among US adults from 1999 to 2018 4](#_Toc132204996)

[sTable 4 Prevalence of previous diagnosed hypertension among US adults from 1999 to 2018 5](#_Toc132204997)

[sTable 5 Annual prevalence change of previous diagnosed hypertension among US adults from 1999 to 2018 7](#_Toc132204998)

[sTable 6 Prevalence of newly diagnosed hypertension among US adults from 1999 to 2018 8](#_Toc132204999)

[sTable 7 Annual prevalence change of newly diagnosed hypertension among US adults from 1999 to 2018 10](#_Toc132205000)

[sTable 8 Prevalence of awareness among US hypertension adults from 1999 to 2018 11](#_Toc132205001)

[sTable 9 Annual prevalence change of awareness among US hypertension adults from 1999 to 2018 13](#_Toc132205002)

[sTable 10 Prevalence of treatment among US hypertension adults from 1999 to 2018 13](#_Toc132205003)

[sTable 11 Annual prevalence change of treatment among US hypertension adults from 1999 to 2018 16](#_Toc132205004)

[sTable 12 Prevalence of control among US hypertension adults from 1999 to 2018 17](#_Toc132205005)

[sTable 13 Annual prevalence change of control among US hypertension adults from 1999 to 2018 19](#_Toc132205006)

[sTable 14 Prevalence of antihypertensive use among US hypertension adults from 1999 to 2018 20](#_Toc132205007)

[sTable 15 Annual prevalence change of antihypertensive use among US hypertension adults from 1999 to 2018 20](#_Toc132205008)


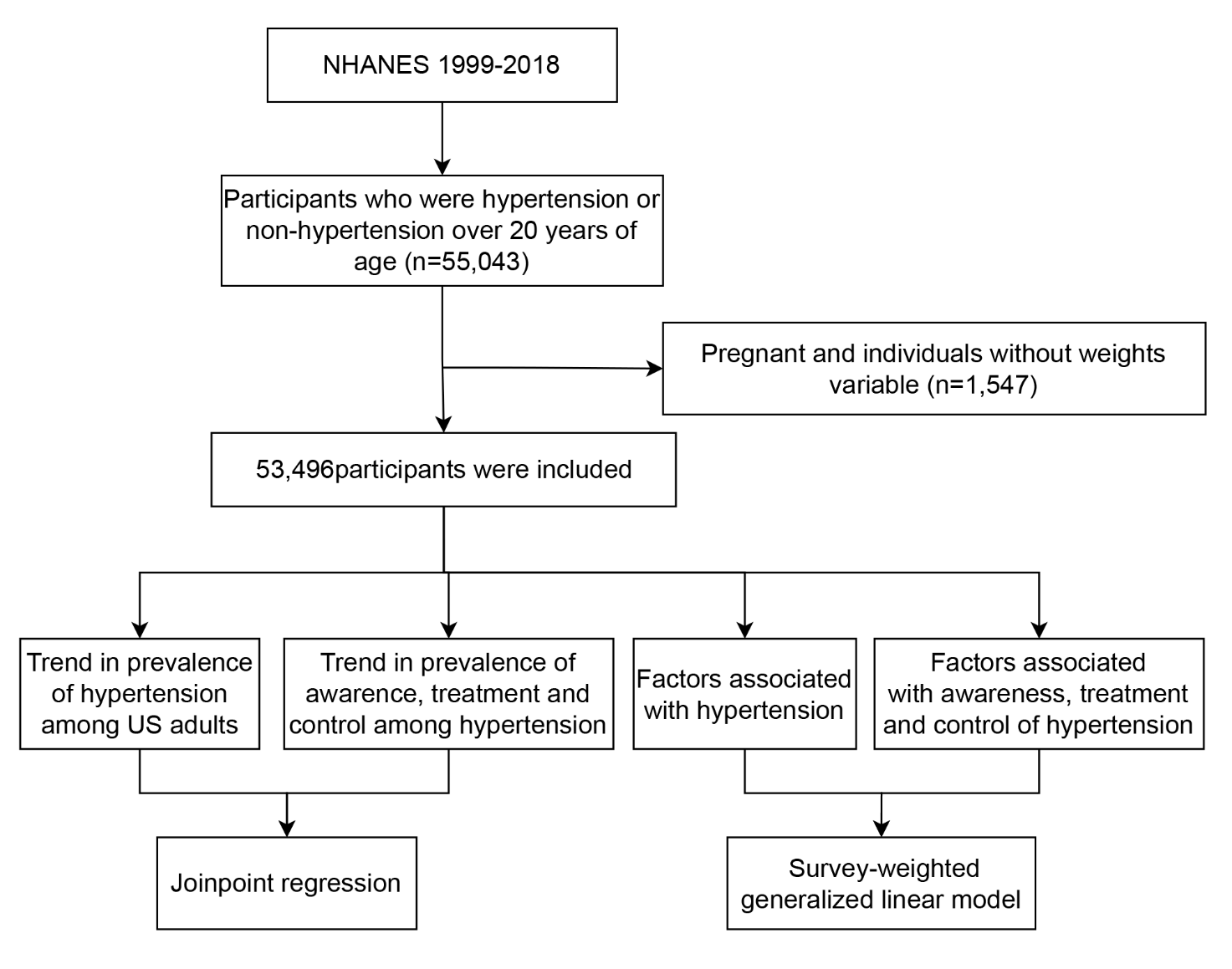


**sFigure 1 Flow chart of this study**

**sTable 1 Classification of antihypertensive drugs among US hypertension adults from 1999 to 2018**

| **ACEI** | **ARB** | **CCB** | **β-blocker** | **Diuretic** |
| --- | --- | --- | --- | --- |
| benazepril | irbesartan | amlodipine | atenolol | hydrochlorothiazide |
| lisinopril | losartan | verapamil | bisoprolol | bendroflumethiazide |
| captopril | olmesartan |  | nadolol | hydroflumethiazide |
| enalapril | valsartan |  | metoprolol | methyclothiazide |
| fosinopril | azilsartan |  | propranolol | hydrochlorothiazide |
| trandolapril | candesartan |  | timolol | chlorthalidone |
| quinapril | eprosartan |  |  | triamterene |
| perindopril | telmisartan |  |  | amiloride |
| moexipril |  |  |  | spironolactone |
| benazepril |  |  |  |  |

ACEI: angiotensin-converting enzyme inhibitors; ARB: angiotensin receptor blockers; CCB: calcium channel blocker.

**sTable 2 Definitions of the individual components of the Life’s simple 7 metric**

| Component | Poor (0 points) | Intermediate (1 point) | Ideal (2 points) |
| --- | --- | --- | --- |
| Blood pressure | Treated blood pressure ≥140/90 mm Hg, and blood pressure ≥140/90 mm Hg | Systolic blood pressure 120 to 139 mm Hg or diastolic blood pressure 80 to 89 mm Hg or treated to <120/80 mm Hg | <120/80 mm Hg, without antihypertensive drugs |
| Total cholesterol | ≥240 mg/dL | 200 to 239 mg/dL or treated to <200 mg/dL | <200 mg/dL, without lipid-lowering medication |
| HbA1c | >6.4% | 5.7% to 6.4% or treated with insulin or oral medication to HbA1c <5.7% | <5.7%, without medication |
| Smoking | Current smoker (smoked at least 100 cigarettes in life and smoked some days or every day now) | Former smoker (smoked at least 100 cigarettes in life and not at all now) | Never smoker (smoked less than 100 cigarettes in life) |
| BMI | ≥30 kg/m^2^ | 25 to 29.9 kg/m^2^ | <25 kg/m^2^ |
| Physical activity | No activity | 1 to 149 minutes moderate/vigorous per week | ≥150 minutes moderate/vigorous per week |
| HEI-2010 ^a^ | <50 | <50 | >80 |

^a^ HEI-2010 score involved 12 components, including total vegetables, greens and beans, total fruits, whole fruits, whole grains, dairy, total protein foods, seafood and plant proteins, fatty acids, sodium, refined grains, sofas calories. BMI: body mass index; HEI: healthy eating index.

**sTable 3 Annual prevalence change of hypertension among US adults from 1999 to 2018**

| **Characteristics** | | **Jointpoint** | **APC1** | ***P*** | **APC2** | ***P*** | **AAPC** | ***P*** |
| --- | --- | --- | --- | --- | --- | --- | --- | --- |
| **Total** |  | 2003 | 2.43(-4.511, 9.876) | 0.419 | 0.702(-0.048, 1.458) | 0.061 | 0.896(0.42, 1.375) | **0.002** |
| **Age (years)** | 20-49 | 2009 | -1.009(-4.177, 2.265) | 0.459 | 2.991(-1.33, 7.502) | 0.137 | 0.728(-0.52, 1.991) | 0.216 |
|  | 40-59 | 2003 | 2.338(-10.651, 17.215) | 0.68 | 0.558(-0.745, 1.877) | 0.323 | 0.749(-0.023, 1.526) | 0.056 |
|  | 60-85 | 2003 | 0.926(-1.375, 3.28) | 0.351 | -0.228(-0.523, 0.068) | 0.104 | -0.05(-0.288, 0.188) | 0.639 |
| **Sex** | Female | 2003 | 1.441(-6.203, 9.709) | 0.658 | 0.347(-0.648, 1.352) | 0.412 | 0.503(-0.011, 1.019) | 0.054 |
|  | Male | 2003 | 3.411(-3.241, 10.519) | 0.251 | 1.006(0.397, 1.619) | **0.008** | 1.231(0.738, 1.726) | **<0.001** |
| **Race** | Non-Hispanic white | 2003 | 3.41(-5.683, 13.378) | 0.392 | 0.642(-0.446, 1.742) | 0.191 | 0.972(0.336, 1.611) | **0.008** |
|  | Non-Hispanic black | 2003 | 2.313(-3.481, 8.455) | 0.36 | 0.736(0.114, 1.362) | **0.029** | 1.003(0.618, 1.39) | **<0.001** |
|  | Mexican American | 2003 | -1.831(-9.107, 6.028) | 0.564 | 2.515(1.212, 3.835) | **0.004** | 1.683(0.415, 2.968) | **0.015** |
|  | Other Hispanic | 2009 | -0.276(-3.616, 3.18) | 0.843 | 2.785(-1.079, 6.799) | 0.125 | 1.31(0.173, 2.46) | **0.029** |
|  | Other race | 2009 | -1.01(-5.417, 3.602) | 0.591 | 3.213(0.103, 6.419) | **0.045** | 1.124(-0.116, 2.379) | 0.07 |
| **Marital status** | No | 2009 | 0.641(-0.857, 2.162) | 0.323 | 1.411(-0.485, 3.344) | 0.115 | 0.984(0.442, 1.529) | **0.003** |
|  | Yes | 2003 | 2.538(-6.698, 12.688) | 0.525 | 0.499(-0.373, 1.38) | 0.202 | 0.685(0.022, 1.352) | **0.044** |
| **Education level** | Less than high school | 2011 | 0.971(-0.232, 2.188) | 0.093 | -0.876(-4.98, 3.405) | 0.616 | 0.578(-0.039, 1.2) | 0.063 |
|  | High school graduate | 2007 | 0.45(-3.726, 4.808) | 0.796 | 1.474(-1.14, 4.156) | 0.209 | 1.084(0.368, 1.806) | **0.008** |
|  | Some college | 2003 | 2.755(-2.395, 8.177) | 0.232 | 1.695(1.013, 2.382) | **0.001** | 1.838(1.422, 2.256) | **<0.001** |
|  | College graduate or above | 2003 | 4.641(-7.739, 18.683) | 0.397 | 0.367(-1.199, 1.957) | 0.576 | 0.995(0.147, 1.849) | **0.027** |
| **PIR** | Low income | 2009 | -0.037(-1.59, 1.54) | 0.954 | 2.967(0.663, 5.324) | **0.021** | 1.286(0.499, 2.078) | **0.005** |
|  | Middle income | 2013 | 1.115(-0.054, 2.297) | 0.058 | -2.407(-9.074, 4.749) | 0.417 | 0.567(-0.109, 1.248) | 0.089 |
|  | High income | 2003 | 3.593(-3.774, 11.524) | 0.273 | 0.479(-0.307, 1.271) | 0.179 | 0.827(0.187, 1.47) | **0.018** |
| **Health insurance** | No | 2003 | 4.582(-6.869, 17.44) | 0.366 | 1.049(-0.036, 2.146) | 0.056 | 1.45(0.674, 2.232) | **0.003** |
|  | Yes | 2003 | 2.316(-5.043, 10.244) | 0.466 | 0.52(-0.272, 1.318) | 0.153 | 0.718(0.235, 1.203) | **0.009** |
| **Life’s simple 7** | Inadequate health | 2011 | 0.776(-0.932, 2.513) | 0.298 | 2.405(-0.892, 5.812) | 0.121 | 1.368(0.805, 1.934) | **<0.001** |
|  | Average health | 2005 | 2.01(-4.37, 8.816) | 0.464 | 0.861(-0.938, 2.693) | 0.275 | 1.168(0.457, 1.883) | **0.005** |
|  | Optimal health | 2003 | 16.933(-23.013, 77.604) | 0.38 | -0.955(-5.077, 3.345) | 0.587 | 0.877(-2.097, 3.941) | 0.52 |

PIR: income-to-poverty ratio; APC1: annual rate of change before inflection; APC2: annual rate of change after inflection; AAPC: average annual rates of change

**sTable 4 Prevalence of previous diagnosed hypertension among US adults from 1999 to 2018**

| **characteristics** | | **Total (n=49705)** | **1999-2000 (n=4107)** | **2001-2002 (n=4604)** | **2003-2004 (n=4428)** | **2005-2006 (n=4315)** | **2007-2008 (n=5482)** | **2009-2010 (n=5801)** | **2011-2012 (n=5184)** | **2013-2014 (n=5409)** | **2015-2016 (n=5307)** | **2017-2018 (n=5068)** | **AAPC** | ***P*** |
| --- | --- | --- | --- | --- | --- | --- | --- | --- | --- | --- | --- | --- | --- | --- |
| **Total** |  | 31.04(29.67, 32.41) | 24.84(22.36, 27.33) | 25.98(23.62, 28.34) | 30.73(28.00, 33.45) | 30.42(28.19, 32.65) | 31.09(28.91, 33.26) | 30.35(27.92, 32.78) | 32.35(29.54, 35.17) | 35.51(33.23, 37.79) | 32.82(30.28, 35.37) | 33.03(30.10, 35.96) | 1.544(0.74, 2.353) | **0.002** |
| **Age (years)** | 20-49 | 11.59(10.89, 12.29) | 8.98(6.56, 11.40) | 10.97(9.04, 12.89) | 11.87(9.70, 14.04) | 10.43(7.77, 13.08) | 11.26(9.27, 13.25) | 10.02(8.29, 11.75) | 10.95(9.13, 12.78) | 14.85(12.63, 17.07) | 12.48(10.42, 14.53) | 13.55(10.81, 16.29) | 1.647(0.053, 3.265) | **0.044** |
|  | 40-59 | 31.89(30.76, 33.01) | 28.66(23.89, 33.43) | 26.83(23.62, 30.04) | 32.15(28.70, 35.60) | 32.40(28.14, 36.65) | 32.84(29.84, 35.85) | 30.25(27.41, 33.10) | 34.03(31.22, 36.83) | 35.57(32.02, 39.13) | 32.71(29.03, 36.39) | 32.54(28.10, 36.98) | 0.989(0.052, 1.935) | **0.041** |
|  | 60-85 | 57.92(56.77, 59.06) | 49.24(46.21, 52.27) | 52.92(49.39, 56.46) | 59.36(55.65, 63.07) | 58.32(56.98, 59.67) | 58.90(55.98, 61.82) | 60.09(56.79, 63.39) | 59.04(55.35, 62.74) | 62.42(59.54, 65.30) | 58.47(54.15, 62.80) | 57.13(52.45, 61.81) | 0.781(0.033, 1.535) | **0.043** |
| **Sex** | Female | 31.99(31.06, 32.91) | 26.62(23.94, 29.31) | 28.51(25.38, 31.64) | 31.46(27.66, 35.26) | 32.16(29.32, 35.01) | 32.25(29.69, 34.81) | 30.87(28.57, 33.17) | 32.84(30.13, 35.55) | 36.88(33.88, 39.88) | 33.26(29.94, 36.57) | 31.53(28.68, 34.37) | 1.043(0.134, 1.961) | **0.029** |
|  | Male | 30.06(29.09, 31.03) | 22.99(19.78, 26.19) | 23.32(20.45, 26.19) | 29.95(26.79, 33.11) | 28.62(26.10, 31.13) | 29.86(26.59, 33.14) | 29.80(26.54, 33.05) | 31.83(28.39, 35.27) | 34.06(31.59, 36.52) | 32.37(29.68, 35.06) | 34.62(31.10, 38.14) | 1.953(1.13, 2.783) | **0.001** |
| **Race** | Non-Hispanic white | 31.97(30.98, 32.97) | 24.89(22.05, 27.73) | 26.66(24.00, 29.32) | 32.03(28.74, 35.33) | 31.43(28.65, 34.21) | 32.47(29.42, 35.53) | 30.89(27.71, 34.07) | 33.21(29.51, 36.91) | 37.94(34.86, 41.02) | 33.35(30.08, 36.62) | 34.23(30.43, 38.02) | 1.696(0.684, 2.718) | **0.005** |
|  | Non-Hispanic black | 39.28(38.19, 40.38) | 31.42(27.58, 35.27) | 36.16(32.12, 40.20) | 35.70(32.29, 39.12) | 38.47(35.83, 41.12) | 38.10(34.92, 41.28) | 40.57(36.88, 44.25) | 41.86(39.09, 44.64) | 43.46(40.12, 46.80) | 40.81(37.56, 44.07) | 41.05(36.71, 45.38) | 1.329(0.668, 1.994) | **0.002** |
|  | Mexican American | 19.96(18.63, 21.30) | 17.05(14.09, 20.02) | 12.50(10.77, 14.23) | 18.72(12.92, 24.51) | 17.41(14.48, 20.34) | 18.16(15.45, 20.86) | 20.28(16.95, 23.61) | 22.50(17.18, 27.83) | 22.89(19.25, 26.53) | 25.87(20.30, 31.43) | 19.63(16.87, 22.39) | 2.56(0.719, 4.434) | **0.012** |
|  | Other Hispanic | 24.48(22.56, 26.40) | 22.38(15.99, 28.76) | 19.46(14.67, 24.26) | 23.17(11.24, 35.09) | 17.90(8.78, 27.03) | 24.11(19.61, 28.61) | 23.96(21.23, 26.69) | 27.26(21.69, 32.83) | 24.99(22.08, 27.89) | 27.01(22.35, 31.67) | 27.59(21.64, 33.54) | 1.643(0.731, 2.564) | **0.003** |
|  | Other race | 26.85(24.87, 28.83) | 23.65(15.91, 31.39) | 19.78(13.21, 26.35) | 24.75(19.30, 30.20) | 26.24(19.12, 33.36) | 25.16(16.01, 34.32) | 25.22(19.39, 31.04) | 25.03(19.96, 30.11) | 26.02(21.13, 30.91) | 30.10(24.32, 35.88) | 31.98(27.15, 36.81) | 1.886(0.914, 2.866) | **0.002** |
| **Marital status** | No | 29.77(28.79, 30.76) | 22.68(20.24, 25.12) | 26.64(23.91, 29.38) | 27.85(24.18, 31.51) | 29.44(25.63, 33.26) | 28.00(25.50, 30.51) | 27.69(25.10, 30.29) | 31.12(27.98, 34.27) | 34.62(31.30, 37.95) | 33.07(29.91, 36.23) | 31.13(28.19, 34.06) | 1.731(0.868, 2.602) | **0.002** |
|  | Yes | 32.21(31.26, 33.15) | 27.26(23.77, 30.76) | 25.53(22.56, 28.49) | 32.86(30.02, 35.71) | 31.10(28.99, 33.20) | 33.47(30.79, 36.14) | 32.36(29.70, 35.03) | 33.45(29.83, 37.07) | 36.21(33.40, 39.02) | 32.62(29.37, 35.87) | 34.71(31.34, 38.08) | 1.245(0.304, 2.195) | **0.016** |
| **Education level** | Less than high school | 36.08(34.85, 37.31) | 30.32(27.46, 33.19) | 33.38(28.09, 38.67) | 35.92(30.65, 41.19) | 35.10(32.01, 38.20) | 35.93(32.50, 39.35) | 36.54(33.13, 39.95) | 40.90(38.23, 43.56) | 38.23(34.79, 41.68) | 37.46(33.57, 41.35) | 36.35(30.91, 41.80) | 1.34(0.461, 2.226) | **0.008** |
|  | High school graduate | 33.54(32.35, 34.73) | 27.53(22.08, 32.99) | 26.79(22.60, 30.98) | 33.58(31.38, 35.79) | 32.56(28.86, 36.26) | 32.79(29.96, 35.62) | 34.37(31.28, 37.46) | 34.26(29.44, 39.08) | 39.16(36.72, 41.59) | 37.74(33.51, 41.98) | 34.80(31.00, 38.61) | 1.412(0.427, 2.406) | **0.011** |
|  | Some college | 31.28(30.12, 32.45) | 22.81(20.17, 25.45) | 24.47(22.45, 26.50) | 29.07(25.40, 32.74) | 28.15(24.61, 31.69) | 29.24(26.53, 31.96) | 30.63(28.15, 33.11) | 32.03(26.59, 37.48) | 38.60(34.88, 42.33) | 34.70(30.70, 38.71) | 36.73(32.72, 40.74) | 2.767(1.991, 3.548) | **<0.001** |
|  | College graduate or above | 25.41(24.12, 26.69) | 18.20(14.10, 22.30) | 21.35(17.54, 25.16) | 25.46(20.72, 30.20) | 27.74(23.45, 32.04) | 27.56(24.08, 31.05) | 22.47(17.91, 27.03) | 26.79(23.06, 30.52) | 28.12(25.22, 31.01) | 25.65(21.30, 29.99) | 26.53(22.27, 30.78) | 1.256(-0.185, 2.717) | 0.08 |
| **PIR** | Low income | 30.28(28.82, 31.73) | 26.44(21.70, 31.18) | 27.50(23.57, 31.44) | 26.86(22.78, 30.94) | 27.05(22.79, 31.30) | 30.05(26.55, 33.55) | 29.02(25.72, 32.32) | 29.50(23.78, 35.22) | 34.03(31.14, 36.92) | 35.25(29.63, 40.87) | 32.46(28.15, 36.77) | 1.633(0.934, 2.336) | **0.001** |
|  | Middle income | 34.41(33.26, 35.57) | 28.60(25.49, 31.71) | 29.65(26.63, 32.68) | 33.20(28.15, 38.24) | 35.22(32.94, 37.49) | 32.06(28.83, 35.29) | 34.22(30.88, 37.56) | 36.74(32.65, 40.84) | 40.22(36.89, 43.54) | 36.82(33.66, 39.98) | 33.28(29.30, 37.27) | 1.306(0.277, 2.345) | **0.019** |
|  | High income | 30.36(29.42, 31.29) | 23.60(20.67, 26.54) | 24.81(22.08, 27.53) | 30.97(27.92, 34.02) | 29.61(27.03, 32.18) | 31.03(28.55, 33.51) | 29.98(26.83, 33.13) | 31.79(28.48, 35.09) | 34.41(31.29, 37.52) | 30.93(28.01, 33.86) | 33.28(29.98, 36.59) | 1.466(0.504, 2.437) | **0.008** |
| **Health insurance** | No | 19.04(17.91, 20.17) | 15.28(12.64, 17.92) | 15.58(12.14, 19.02) | 18.04(14.57, 21.50) | 19.23(14.55, 23.91) | 17.21(14.97, 19.44) | 18.09(15.30, 20.88) | 20.74(17.27, 24.21) | 20.66(17.91, 23.41) | 22.65(18.13, 27.18) | 22.40(17.75, 27.04) | 2.187(1.446, 2.933) | **<0.001** |
|  | Yes | 33.66(32.76, 34.56) | 27.13(24.04, 30.21) | 28.32(25.85, 30.79) | 33.66(30.68, 36.64) | 32.99(30.55, 35.43) | 34.43(31.99, 36.86) | 33.59(31.19, 36.00) | 35.23(31.82, 38.63) | 38.74(35.98, 41.51) | 34.32(31.20, 37.44) | 34.75(31.61, 37.89) | 1.323(0.385, 2.269) | **0.012** |
| **Life’s simple 7** | Inadequate health | 58.16(56.33, 59.99) | 49.17(42.74, 55.59) | 45.67(37.09, 54.25) | 56.44(52.03, 60.85) | 51.48(44.64, 58.32) | 57.55(51.20, 63.89) | 61.70(56.17, 67.23) | 59.52(55.48, 63.55) | 62.30(58.54, 66.05) | 68.07(63.12, 73.02) | 65.11(60.41, 69.82) | 1.591(0.947, 2.239) | **<0.001** |
|  | Average health | 35.53(34.63, 36.44) | 28.16(25.90, 30.41) | 29.50(26.75, 32.25) | 33.07(29.47, 36.67) | 34.73(32.27, 37.19) | 37.04(34.52, 39.56) | 36.72(34.24, 39.20) | 39.72(36.53, 42.91) | 35.35(32.34, 38.36) | 39.17(36.41, 41.93) | 39.59(36.63, 42.55) | 1.724(0.949, 2.506) | **0.001** |
|  | Optimal health | 10.09(9.32, 10.86) | 5.66(3.50, 7.82) | 7.72(5.70, 9.74) | 11.94(9.35, 14.54) | 10.96(8.51, 13.41) | 11.14(9.38, 12.90) | 9.90(7.68, 12.13) | 11.59(9.30, 13.89) | 6.31(3.96, 8.65) | 10.47(8.21, 12.74) | 11.03(7.88, 14.18) | 1.003(-2.02, 4.12) | 0.471 |

PIR: income-to-poverty ratio; AAPC: average annual percent change

**sTable 5 Annual prevalence change of previous diagnosed hypertension among US adults from 1999 to 2018**

| **Characteristics** | | **Jointpoint** | **APC1** | ***P*** | **APC2** | ***P*** | **AAPC** | ***P*** |
| --- | --- | --- | --- | --- | --- | --- | --- | --- |
| **Total** |  | 2003 | 5.398(-5.313, 17.321) | 0.263 | 0.99(-0.177, 2.171) | 0.081 | 1.544(0.74, 2.353) | **0.002** |
| **Age (years)** | 20-49 | 2009 | 0.323(-5.259, 6.234) | 0.891 | 3.344(-3.621, 10.812) | 0.28 | 1.647(0.053, 3.265) | **0.044** |
|  | 40-59 | 2013 | 1.456(-0.203, 3.142) | 0.074 | -1.996(-14.71, 12.613) | 0.724 | 0.989(0.052, 1.935) | **0.041** |
|  | 60-85 | 2003 | 4.461(-2.199, 11.574) | 0.149 | 0.255(-0.378, 0.892) | 0.348 | 0.781(0.033, 1.535) | **0.043** |
| **Sex** | Female | 2013 | 1.749(0.565, 2.948) | **0.013** | -2.724(-11.099, 6.44) | 0.466 | 1.043(0.134, 1.961) | **0.029** |
|  | Male | 2003 | 6.605(-2.214, 16.219) | 0.115 | 1.403(0.572, 2.24) | **0.007** | 1.953(1.13, 2.783) | **0.001** |
| **Race** | Non-Hispanic white | 2003 | 6.302(-6.991, 21.496) | 0.293 | 0.98(-0.636, 2.623) | 0.181 | 1.696(0.684, 2.718) | **0.005** |
|  | Non-Hispanic black | 2013 | 1.871(1.079, 2.67) | **0.002** | -1.899(-7.574, 4.125) | 0.446 | 1.329(0.668, 1.994) | **0.002** |
|  | Mexican American | 2013 | 3.866(0.202, 7.663) | **0.042** | -3.495(-26.444, 26.613) | 0.75 | 2.56(0.719, 4.434) | **0.012** |
|  | Other Hispanic | 2003 | 0.486(-13.928, 17.314) | 0.939 | 1.837(0.167, 3.536) | **0.037** | 1.643(0.731, 2.564) | **0.003** |
|  | Other race | 2013 | 0.965(-0.8, 2.761) | 0.221 | 5.293(-3.571, 14.972) | 0.192 | 1.886(0.914, 2.866) | **0.002** |
| **Marital status** | No | 2013 | 2.295(0.818, 3.794) | **0.01** | -1.141(-10.618, 9.341) | 0.782 | 1.731(0.868, 2.602) | **0.002** |
|  | Yes | 2003 | 5.359(-6.107, 18.225) | 0.297 | 0.785(-0.263, 1.843) | 0.112 | 1.245(0.304, 2.195) | **0.016** |
| **Education level** | Less than high school | 2011 | 2.2(1.295, 3.112) | **0.001** | -1.677(-4.685, 1.427) | 0.221 | 1.34(0.461, 2.226) | **0.008** |
|  | High school graduate | 2013 | 1.975(0.034, 3.954) | **0.047** | -2.131(-13.491, 10.721) | 0.672 | 1.412(0.427, 2.406) | **0.011** |
|  | Some college | 2013 | 3.255(2.162, 4.36) | **0.001** | -0.074(-7.931, 8.454) | 0.982 | 2.767(1.991, 3.548) | **<0.001** |
|  | College graduate or above | 2003 | 10.355(-7.347, 31.438) | 0.207 | 0.117(-1.605, 1.869) | 0.869 | 1.256(-0.185, 2.717) | 0.08 |
| **PIR** | Low income | 2013 | 1.894(0.989, 2.808) | **0.003** | 0.057(-5.975, 6.475) | 0.982 | 1.633(0.934, 2.336) | **0.001** |
|  | Middle income | 2013 | 2.143(0.528, 3.784) | **0.019** | -3.823(-14.303, 7.939) | 0.425 | 1.306(0.277, 2.345) | **0.019** |
|  | High income | 2003 | 6.89(-3.269, 18.115) | 0.147 | 0.752(-0.288, 1.803) | 0.122 | 1.466(0.504, 2.437) | **0.008** |
| **Health insurance** | No | 2007 | 1.586(-1.049, 4.291) | 0.184 | 2.653(0.987, 4.347) | **0.009** | 2.187(1.446, 2.933) | **<0.001** |
|  | Yes | 2013 | 2.038(0.571, 3.527) | **0.016** | -2.674(-12.394, 8.124) | 0.537 | 1.323(0.385, 2.269) | **0.012** |
|  | Obesity | 2013 | 1.247(0.453, 2.047) | **0.01** | -2.222(-6.776, 2.555) | 0.28 | 0.704(0.118, 1.294) | **0.024** |
| **Life’s simple 7** | Inadequate health | 2003 | 3.37(-10.832, 19.834) | 0.589 | 1.419(0.268, 2.584) | **0.025** | 1.591(0.947, 2.239) | **<0.001** |
|  | Average health | 2007 | 3.471(1.073, 5.926) | **0.013** | 0.58(-0.968, 2.151) | 0.382 | 1.724(0.949, 2.506) | **0.001** |
|  | Optimal health | 2003 | 19.965(-19.831, 79.516) | 0.298 | -0.965(-4.665, 2.879) | 0.542 | 1.003(-2.02, 4.12) | 0.471 |

PIR: income-to-poverty ratio; APC1: annual rate of change before inflection; APC2: annual rate of change after inflection; AAPC: average annual rates of change

**sTable 6 Prevalence of newly diagnosed hypertension among US adults from 1999 to 2018**

|  | | | | | | | | | | | | |  |  |
| --- | --- | --- | --- | --- | --- | --- | --- | --- | --- | --- | --- | --- | --- | --- |
| **Characteristics** | | **Total (n=34304)** | **1999-2000 (n=3095)** | **2001-2002 (n=3453)** | **2003-2004 (n=3036)** | **2005-2006 (n=3063)** | **2007-2008 (n=3736)** | **2009-2010 (n=3944)** | **2011-2012 (n=3487)** | **2013-2014 (n=3547)** | **2015-2016 (n=3569)** | **2017-2018 (n=3374)** | **AAPC** | ***P*** |
| **Total** |  | 6.48(6.10, 6.87) | 8.62(7.23, 10.01) | 8.00(7.26, 8.75) | 6.88(5.54, 8.22) | 6.40(5.38, 7.43) | 5.63(5.07, 6.19) | 5.36(4.32, 6.41) | 5.70(4.84, 6.56) | 4.82(4.06, 5.58) | 5.92(5.01, 6.83) | 7.51(5.99, 9.04) | -2.301(-4.213, -0.352) | **0.026** |
| **Age (years)** | 20-49 | 3.34(2.97, 3.70) | 4.19(2.26, 6.12) | 4.19(3.30, 5.09) | 3.67(1.93, 5.41) | 3.41(2.43, 4.39) | 2.59(1.43, 3.75) | 3.29(2.58, 4.00) | 3.46(2.52, 4.40) | 2.21(1.56, 2.85) | 2.66(1.97, 3.36) | 3.71(2.53, 4.89) | -2.238(-4.415, -0.012) | **0.049** |
|  | 40-59 | 6.60(6.06, 7.14) | 8.07(6.00, 10.15) | 7.18(5.89, 8.48) | 7.41(5.26, 9.56) | 6.57(5.15, 7.98) | 5.68(4.36, 7.00) | 4.56(3.23, 5.88) | 5.93(4.30, 7.57) | 5.73(4.23, 7.23) | 6.85(5.47, 8.22) | 8.45(5.75, 11.15) | -0.71(-2.792, 1.417) | 0.461 |
|  | 60-85 | 10.86(10.17, 11.55) | 17.96(15.48, 20.43) | 16.81(15.12, 18.50) | 11.25(8.64, 13.87) | 10.81(8.67, 12.95) | 10.25(8.50, 12.00) | 9.64(7.15, 12.13) | 8.40(6.56, 10.24) | 6.99(5.66, 8.31) | 8.82(6.80, 10.83) | 10.99(8.33, 13.65) | -4.796(-6.897, -2.648) | **0.001** |
| **Sex** | Female | 5.61(5.19, 6.03) | 7.86(6.38, 9.34) | 7.40(5.92, 8.89) | 6.76(5.29, 8.23) | 4.81(3.66, 5.96) | 4.68(3.88, 5.47) | 4.38(3.31, 5.44) | 4.46(3.22, 5.71) | 3.67(2.67, 4.67) | 4.77(3.68, 5.86) | 7.15(5.16, 9.14) | -2.734(-5.492, 0.104) | 0.057 |
|  | Male | 7.41(6.96, 7.86) | 9.41(7.90, 10.92) | 8.64(7.60, 9.67) | 7.00(5.16, 8.84) | 8.06(6.46, 9.65) | 6.64(5.49, 7.78) | 6.40(5.27, 7.53) | 7.01(5.99, 8.02) | 6.03(4.86, 7.20) | 7.13(5.87, 8.40) | 7.90(5.88, 9.91) | -1.766(-3.111, -0.402) | **0.018** |
| **Race** | Non-Hispanic white | 6.64(6.19, 7.08) | 8.67(7.19, 10.16) | 8.23(7.38, 9.08) | 7.08(5.40, 8.76) | 6.43(5.21, 7.66) | 5.66(5.13, 6.18) | 5.81(4.39, 7.22) | 5.84(4.62, 7.07) | 4.76(3.63, 5.88) | 5.99(4.59, 7.40) | 7.62(5.32, 9.92) | -2.809(-4.828, -0.746) | **0.014** |
|  | Non-Hispanic black | 6.82(6.27, 7.38) | 8.66(5.80, 11.53) | 8.13(6.33, 9.92) | 7.91(5.27, 10.56) | 7.20(5.77, 8.64) | 6.33(4.28, 8.38) | 4.58(2.76, 6.41) | 6.10(4.89, 7.30) | 5.39(4.27, 6.51) | 5.97(4.97, 6.98) | 8.32(6.89, 9.75) | -0.945(-3.219, 1.382) | 0.373 |
|  | Mexican American | 5.85(5.29, 6.42) | 7.50(5.48, 9.53) | 6.57(4.83, 8.32) | 4.93(3.17, 6.68) | 5.72(3.29, 8.15) | 5.99(4.32, 7.67) | 5.24(4.31, 6.16) | 4.61(2.85, 6.37) | 4.58(3.31, 5.85) | 5.68(4.21, 7.16) | 7.86(5.37, 10.36) | -0.895(-3.204, 1.469) | 0.405 |
|  | Other Hispanic | 5.53(4.47, 6.59) | 7.01(1.76, 12.25) | 7.59(4.95, 10.23) | 6.05(0.78, 11.32) | 4.75(0.37, 9.13) | 5.27(3.73, 6.81) | 3.04(1.96, 4.12) | 3.92(2.71, 5.13) | 4.17(2.57, 5.78) | 5.17(2.68, 7.66) | 7.04(4.63, 9.46) | -1.304(-5.725, 3.324) | 0.527 |
|  | Other race | 5.98(5.06, 6.90) | 12.33(5.06, 19.59) | 6.81(1.81, 11.81) | 5.28(2.50, 8.05) | 6.35(3.42, 9.28) | 3.76(-0.29, 7.80) | 4.16(1.52, 6.81) | 6.44(3.83, 9.06) | 5.25(3.50, 7.00) | 6.09(4.47, 7.71) | 6.00(4.12, 7.88) | -1.415(-4.165, 1.413) | 0.279 |
| **Marital status** | No | 6.25(5.84, 6.66) | 8.70(6.88, 10.51) | 7.53(6.46, 8.60) | 6.70(5.61, 7.79) | 6.41(5.04, 7.79) | 6.21(5.23, 7.19) | 5.26(4.08, 6.43) | 5.37(4.22, 6.52) | 4.08(3.38, 4.77) | 5.94(4.56, 7.32) | 6.83(5.25, 8.41) | -2.61(-4.695, -0.479) | **0.023** |
|  | Yes | 6.64(6.17, 7.12) | 8.75(7.24, 10.25) | 8.33(7.18, 9.49) | 7.03(5.00, 9.05) | 6.35(5.13, 7.58) | 5.19(4.06, 6.31) | 5.46(3.90, 7.01) | 6.00(4.54, 7.45) | 5.42(4.19, 6.66) | 5.91(4.65, 7.17) | 8.12(6.09, 10.15) | -1.958(-3.999, 0.126) | 0.062 |
| **Education level** | Less than high school | 7.52(6.98, 8.06) | 9.01(7.05, 10.97) | 9.94(9.10, 10.78) | 9.01(7.60, 10.42) | 8.25(5.71, 10.79) | 6.75(5.27, 8.23) | 5.73(4.19, 7.27) | 5.93(4.33, 7.53) | 5.43(3.76, 7.09) | 6.22(4.85, 7.59) | 8.00(6.35, 9.65) | -2.734(-4.497, -0.937) | **0.008** |
|  | High school graduate | 7.35(6.68, 8.02) | 10.00(7.94, 12.06) | 8.52(6.55, 10.49) | 7.57(5.75, 9.39) | 6.48(4.98, 7.98) | 6.17(4.82, 7.52) | 5.01(3.69, 6.34) | 6.85(4.28, 9.41) | 6.57(4.24, 8.90) | 6.97(5.49, 8.45) | 8.62(5.33, 11.91) | -1.757(-4.096, 0.638) | 0.128 |
|  | Some college | 5.84(5.30, 6.38) | 7.79(6.21, 9.36) | 6.94(5.75, 8.12) | 5.98(3.94, 8.02) | 5.87(4.21, 7.52) | 5.99(4.12, 7.87) | 5.56(3.96, 7.15) | 5.52(3.78, 7.26) | 3.32(2.37, 4.26) | 5.83(3.97, 7.68) | 6.09(4.11, 8.07) | -2.513(-4.609, -0.371) | **0.027** |
|  | College graduate or above | 5.76(5.16, 6.36) | 7.52(4.59, 10.44) | 7.27(5.43, 9.12) | 5.54(3.22, 7.87) | 5.70(3.32, 8.07) | 3.77(2.27, 5.26) | 5.06(3.96, 6.16) | 5.00(3.39, 6.61) | 4.89(3.74, 6.03) | 5.21(3.97, 6.45) | 7.72(5.18, 10.26) | -1.202(-3.822, 1.488) | 0.329 |
| **PIR** | Low income | 5.76(5.02, 6.50) | 8.08(4.53, 11.64) | 6.56(4.28, 8.84) | 6.04(4.66, 7.42) | 6.41(3.31, 9.51) | 4.58(3.15, 6.01) | 4.42(2.90, 5.95) | 4.12(3.11, 5.14) | 4.88(3.33, 6.42) | 4.72(3.17, 6.27) | 8.76(4.52, 13.00) | -1.999(-4.924, 1.016) | 0.163 |
|  | Middle income | 6.61(5.98, 7.23) | 8.95(5.78, 12.11) | 7.65(5.52, 9.77) | 6.80(4.84, 8.76) | 7.62(5.25, 10.00) | 7.61(6.11, 9.11) | 6.27(4.39, 8.16) | 5.52(3.50, 7.53) | 4.36(3.30, 5.42) | 5.12(4.05, 6.20) | 6.47(5.08, 7.87) | -2.508(-4.513, -0.461) | **0.023** |
|  | High income | 6.50(6.08, 6.92) | 8.31(7.06, 9.56) | 8.28(7.06, 9.50) | 6.97(5.20, 8.73) | 6.19(5.11, 7.28) | 5.33(4.49, 6.17) | 5.30(4.30, 6.29) | 6.30(4.95, 7.65) | 5.00(4.07, 5.92) | 6.21(4.85, 7.57) | 7.13(5.30, 8.95) | -2.242(-4.214, -0.23) | **0.033** |
| **Health insurance** | No | 5.68(5.08, 6.28) | 4.73(2.49, 6.97) | 5.13(3.23, 7.03) | 6.60(4.45, 8.75) | 5.69(3.10, 8.27) | 7.50(6.19, 8.81) | 5.00(3.44, 6.57) | 4.99(3.49, 6.50) | 5.54(4.13, 6.96) | 4.50(3.48, 5.53) | 6.75(3.90, 9.60) | -1.289(-4.289, 1.805) | 0.361 |
|  | Yes | 6.64(6.27, 7.02) | 9.49(7.98, 10.99) | 8.59(7.64, 9.55) | 6.87(5.43, 8.31) | 6.53(5.46, 7.59) | 5.18(4.62, 5.74) | 5.46(4.45, 6.48) | 5.88(4.92, 6.84) | 4.67(3.89, 5.45) | 6.15(5.03, 7.26) | 7.65(5.92, 9.37) | -2.806(-5.373, -0.168) | **0.04** |
| **Life’s simple 7** | Inadequate health | 13.37(12.25, 14.49) | 19.42(14.70, 24.13) | 17.66(14.19, 21.14) | 11.38(7.56, 15.20) | 13.32(10.47, 16.18) | 13.26(10.61, 15.91) | 9.73(7.05, 12.40) | 11.97(9.25, 14.69) | 10.57(8.58, 12.55) | 13.47(9.11, 17.84) | 15.06(8.93, 21.18) | -2.798(-5.031, -0.513) | **0.023** |
|  | Average health | 7.64(7.21, 8.06) | 9.65(8.14, 11.16) | 9.08(8.13, 10.04) | 8.05(6.61, 9.49) | 7.38(6.02, 8.75) | 6.56(5.62, 7.49) | 7.02(5.51, 8.53) | 7.29(5.81, 8.77) | 4.49(3.48, 5.49) | 7.29(5.97, 8.61) | 9.61(7.93, 11.28) | -1.356(-3.7, 1.045) | 0.227 |
|  | Optimal health | 1.13(0.88, 1.38) | 1.47(0.39, 2.55) | 0.93(0.23, 1.63) | 1.14(0.35, 1.94) | 1.20(0.86, 1.53) | 1.48(0.71, 2.26) | 1.01(0.55, 1.47) | 1.10(0.36, 1.84) | 0.11(-0.10, 0.32) | 1.11(0.38, 1.84) | 1.20(0.16, 2.24) | -1.265(-5.627, 3.299) | 0.534 |

PIR: income-to-poverty ratio; AAPC: average annual percent change

**sTable 7 Annual prevalence change of newly diagnosed hypertension among US adults from 1999 to 2018**

| **Characteristics** | | **Jointpoint** | **APC1** | ***P*** | **APC2** | ***P*** | **AAPC** | ***P*** |
| --- | --- | --- | --- | --- | --- | --- | --- | --- |
| **Total** |  | 2013 | -4.075(-5.682, -2.442) | **0.001** | 12.302(-4.543, 32.118) | 0.126 | -2.301(-4.213, -0.352) | **0.026** |
| **Age** | 20~ | 2013 | -3.75(-6.142, -1.297) | **0.011** | 6.989(-11.618, 29.514) | 0.405 | -2.238(-4.415, -0.012) | **0.049** |
|  | 40~ | 2009 | -4.821(-7.231, -2.348) | **0.004** | 5.591(0.976, 10.418) | **0.026** | -0.71(-2.792, 1.417) | 0.461 |
|  | 60~ | 2013 | -6.669(-8.819, -4.468) | **0.001** | 12.358(-11.477, 42.61) | 0.265 | -4.796(-6.897, -2.648) | **0.001** |
| **Sex** | Female | 2013 | -5.638(-7.741, -3.487) | **0.001** | 18.749(-2.378, 44.448) | 0.074 | -2.734(-5.492, 0.104) | 0.057 |
|  | Male | 2009 | -3.7(-6.718, -0.585) | **0.029** | 1.86(-3.309, 7.305) | 0.405 | -1.766(-3.111, -0.402) | **0.018** |
| **Race** | Non-Hispanic white | 2013 | -4.409(-6.224, -2.558) | **0.002** | 13.596(-10.391, 44.006) | 0.226 | -2.809(-4.828, -0.746) | **0.014** |
|  | Non-Hispanic black | 2013 | -3.668(-5.852, -1.434) | **0.009** | 11.206(-0.789, 24.651) | 0.062 | -0.945(-3.219, 1.382) | 0.373 |
|  | Mexican American | 2013 | -3.086(-5.254, -0.869) | **0.016** | 13.886(-5.092, 36.658) | 0.126 | -0.895(-3.204, 1.469) | 0.405 |
|  | Other Hispanic | 2009 | -9.158(-12.37, -5.829) | **0.001** | 7.914(3.647, 12.357) | **0.005** | -1.304(-5.725, 3.324) | 0.527 |
|  | Other race | 2003 | -18.017(-47.082, 27.012) | 0.296 | 0.845(-2.867, 4.698) | 0.589 | -1.415(-4.165, 1.413) | 0.279 |
| **Marital status** | No | 2013 | -4.467(-5.591, -3.33) | **<0.001** | 12.206(0.929, 24.743) | **0.038** | -2.61(-4.695, -0.479) | **0.023** |
|  | Yes | 2009 | -5.806(-8.996, -2.504) | **0.007** | 4.59(-1.764, 11.356) | 0.125 | -1.958(-3.999, 0.126) | 0.062 |
| **Education level** | Less than high school | 2013 | -5.048(-7.199, -2.847) | **0.002** | 11.043(-6.936, 32.494) | 0.188 | -2.734(-4.497, -0.937) | **0.008** |
|  | High school graduate | 2009 | -6.05(-7.809, -4.258) | **<0.001** | 5.592(0.949, 10.449) | **0.027** | -1.757(-4.096, 0.638) | 0.128 |
|  | Some college | 2013 | -4.183(-5.399, -2.95) | **<0.001** | 11.025(-1.876, 25.623) | 0.081 | -2.513(-4.609, -0.371) | **0.027** |
|  | College graduate or above | 2007 | -7.555(-16.695, 2.587) | 0.11 | 3.61(-1.314, 8.779) | 0.12 | -1.202(-3.822, 1.488) | 0.329 |
| **PIR** | Low income | 2011 | -5.29(-9.274, -1.131) | **0.023** | 9.517(-4.582, 25.698) | 0.151 | -1.999(-4.924, 1.016) | 0.163 |
|  | Middle income | 2013 | -4.45(-7.044, -1.783) | **0.008** | 6.111(-7.48, 21.698) | 0.317 | -2.508(-4.513, -0.461) | **0.023** |
|  | High income | 2007 | -6.075(-10.807, -1.093) | **0.026** | 1.667(-3.093, 6.661) | 0.416 | -2.242(-4.214, -0.23) | **0.033** |
| **Health insurance** | No | 2007 | 4.159(-7.452, 17.227) | 0.416 | -4.061(-9.757, 1.995) | 0.142 | -1.289(-4.289, 1.805) | 0.361 |
|  | Yes | 2007 | -7.718(-13.657, -1.37) | **0.027** | 2.002(-3.742, 8.089) | 0.42 | -2.806(-5.373, -0.168) | **0.04** |
| **Life’s simple 7** | Inadequate health | 2009 | -5.85(-10.337, -1.139) | **0.025** | 3(-6.644, 13.64) | 0.475 | -2.798(-5.031, -0.513) | **0.023** |
|  | Average health | 2013 | -4.068(-5.905, -2.195) | **0.003** | 15.428(-0.989, 34.568) | 0.061 | -1.356(-3.7, 1.045) | 0.227 |
|  | Optimal health | 2013 | -2.576(-7.259, 2.344) | 0.231 | 6.332(-30.898, 63.621) | 0.729 | -1.265(-5.627, 3.299) | 0.534 |

PIR: income-to-poverty ratio; APC1: annual rate of change before inflection; APC2: annual rate of change after inflection; AAPC: average annual rates of change

**sTable 8 Prevalence of awareness among US hypertension adults from 1999 to 2018**

| **Characteristics** | | **Total (n=22947)** | **1999-2000 (n=1952)** | **2001-2002 (n=2096)** | **2003-2004 (n=2131)** | **2005-2006 (n=1903)** | **2007-2008 (n=2535)** | **2009-2010 (n=2548)** | **2011-2012 (n=2334)** | **2013-2014 (n=2451)** | **2015-2016 (n=2419)** | **2017-2018 (n=2578)** | **AAPC** | ***P*** |
| --- | --- | --- | --- | --- | --- | --- | --- | --- | --- | --- | --- | --- | --- | --- |
| **Total** |  | 81.97(78.35, 85.59) | 73.22(70.28, 76.16) | 75.85(73.73, 77.97) | 80.64(77.14, 84.15) | 81.74(79.30, 84.17) | 83.94(82.07, 85.82) | 84.32(81.91, 86.72) | 84.52(82.07, 86.98) | 87.52(85.49, 89.55) | 84.09(81.65, 86.53) | 81.16(77.83, 84.49) | 0.737(0.241, 1.235) | **0.009** |
| **Age (years)** | 20-49 | 77.33(75.17, 79.49) | 68.19(59.00, 77.37) | 70.85(64.43, 77.27) | 76.05(66.71, 85.38) | 75.12(69.89, 80.35) | 81.12(72.51, 89.73) | 75.11(69.88, 80.33) | 75.07(68.16, 81.98) | 87.07(83.36, 90.77) | 82.40(78.64, 86.16) | 78.50(72.44, 84.56) | 1.028(0.229, 1.833) | **0.018** |
|  | 40-59 | 82.29(80.99, 83.59) | 76.85(72.61, 81.10) | 78.68(75.29, 82.07) | 80.61(76.11, 85.10) | 82.44(78.52, 86.36) | 84.53(80.70, 88.36) | 86.46(83.29, 89.64) | 84.75(80.91, 88.58) | 85.59(81.71, 89.48) | 82.19(78.60, 85.79) | 79.03(73.59, 84.46) | 0.384(-0.104, 0.875) | 0.107 |
|  | 60-85 | 83.33(82.37, 84.29) | 72.00(68.77, 75.24) | 75.31(72.72, 77.91) | 82.35(79.01, 85.68) | 83.14(80.76, 85.51) | 84.28(81.44, 87.12) | 85.21(82.08, 88.35) | 87.08(84.32, 89.83) | 89.24(87.36, 91.12) | 85.99(82.88, 89.09) | 83.50(79.61, 87.39) | 0.952(0.419, 1.487) | **0.003** |
| **Sex** | Female | 84.25(83.18, 85.32) | 76.10(72.55, 79.66) | 78.56(74.03, 83.09) | 80.93(77.14, 84.73) | 85.88(83.09, 88.68) | 86.29(84.15, 88.44) | 86.77(84.04, 89.51) | 87.36(84.12, 90.59) | 90.34(88.20, 92.48) | 86.69(83.45, 89.93) | 81.26(76.79, 85.72) | 0.661(0.09, 1.235) | **0.028** |
|  | Male | 79.73(78.58, 80.87) | 70.02(66.95, 73.09) | 72.65(69.58, 75.72) | 80.33(75.50, 85.15) | 77.41(73.58, 81.25) | 81.43(77.60, 85.26) | 81.80(79.31, 84.30) | 81.63(78.83, 84.43) | 84.51(81.25, 87.76) | 81.44(78.36, 84.52) | 81.07(76.86, 85.28) | 0.858(0.378, 1.339) | **0.003** |
| **Race** | Non-Hispanic white | 82.13(81.05, 83.22) | 72.93(70.02, 75.84) | 75.68(73.09, 78.28) | 80.73(76.53, 84.93) | 82.35(79.52, 85.18) | 84.38(82.29, 86.47) | 83.40(80.19, 86.60) | 84.72(81.41, 88.02) | 88.34(85.61, 91.08) | 84.11(80.30, 87.92) | 81.64(76.89, 86.40) | 0.881(0.352, 1.413) | **0.005** |
|  | Non-Hispanic black | 84.65(83.47, 85.82) | 78.26(70.94, 85.59) | 81.31(78.13, 84.50) | 81.18(75.23, 87.13) | 82.56(79.17, 85.94) | 85.76(81.70, 89.81) | 89.76(85.78, 93.75) | 86.32(84.19, 88.44) | 88.04(85.57, 90.50) | 86.63(84.83, 88.42) | 83.10(79.76, 86.45) | 0.365(-0.037, 0.768) | 0.07 |
|  | Mexican American | 76.56(74.68, 78.45) | 68.78(61.76, 75.81) | 65.31(58.30, 72.31) | 78.61(74.17, 83.05) | 74.47(65.72, 83.22) | 74.35(69.18, 79.52) | 78.94(75.34, 82.55) | 82.28(76.65, 87.92) | 83.06(79.61, 86.51) | 81.07(76.56, 85.57) | 69.70(63.87, 75.53) | 0.639(-0.303, 1.59) | 0.157 |
|  | Other Hispanic | 80.82(77.62, 84.02) | 76.16(61.07, 91.25) | 71.95(63.97, 79.92) | 76.74(63.79, 89.70) | 73.95(52.32, 95.57) | 80.94(75.25, 86.64) | 87.70(84.95, 90.46) | 86.78(83.84, 89.71) | 85.27(81.14, 89.39) | 83.34(76.68, 90.00) | 79.56(71.77, 87.35) | 0.5(-0.567, 1.578) | 0.313 |
|  | Other race | 81.10(78.39, 83.81) | 63.45(51.84, 75.07) | 74.38(59.09, 89.68) | 82.42(73.23, 91.62) | 80.51(70.44, 90.57) | 85.46(70.20, 100.73) | 85.32(76.16, 94.48) | 78.49(68.92, 88.06) | 83.04(77.22, 88.86) | 82.91(78.66, 87.17) | 83.14(79.16, 87.12) | 0.483(-0.144, 1.113) | 0.114 |
| **Marital status** | No | 81.96(80.94, 82.98) | 71.89(67.60, 76.17) | 77.12(75.02, 79.23) | 79.40(75.99, 82.81) | 80.61(76.80, 84.43) | 81.32(79.15, 83.49) | 83.49(80.60, 86.37) | 84.60(81.19, 88.01) | 88.84(86.80, 90.88) | 84.48(81.66, 87.29) | 81.59(78.00, 85.18) | 0.822(0.354, 1.292) | **0.004** |
|  | Yes | 82.24(81.07, 83.40) | 74.16(70.76, 77.55) | 74.98(72.10, 77.86) | 81.40(76.80, 86.01) | 82.61(80.08, 85.14) | 85.72(82.28, 89.16) | 84.84(81.58, 88.10) | 84.45(80.68, 88.23) | 86.52(83.50, 89.54) | 83.75(79.78, 87.72) | 80.81(76.53, 85.09) | 0.682(0.123, 1.244) | **0.023** |
| **Education level** | Less than high school | 81.86(80.71, 83.02) | 75.92(71.36, 80.49) | 76.54(72.73, 80.34) | 78.39(74.92, 81.85) | 80.24(75.23, 85.26) | 83.11(80.16, 86.05) | 85.78(82.64, 88.92) | 86.69(83.50, 89.88) | 86.39(82.88, 89.90) | 85.30(82.56, 88.05) | 81.34(77.70, 84.97) | 0.605(0.137, 1.075) | **0.017** |
|  | High school graduate | 81.34(79.86, 82.82) | 72.33(65.96, 78.69) | 75.49(70.45, 80.52) | 80.55(76.82, 84.28) | 82.32(78.76, 85.88) | 82.67(79.73, 85.60) | 87.14(84.19, 90.09) | 82.97(77.37, 88.58) | 85.40(80.88, 89.93) | 83.46(80.41, 86.51) | 79.99(73.38, 86.60) | 0.484(-0.11, 1.082) | 0.097 |
|  | Some college | 83.78(82.38, 85.18) | 73.43(69.33, 77.53) | 77.09(73.60, 80.58) | 82.76(77.02, 88.50) | 81.97(76.78, 87.17) | 82.76(78.10, 87.42) | 83.63(79.57, 87.70) | 85.03(80.08, 89.99) | 91.57(89.91, 93.23) | 85.47(80.56, 90.37) | 85.55(81.66, 89.45) | 1.115(0.521, 1.713) | **0.002** |
|  | College graduate or above | 80.80(79.05, 82.54) | 70.15(59.65, 80.66) | 73.96(68.12, 79.80) | 80.23(72.95, 87.51) | 82.09(75.93, 88.25) | 87.97(83.36, 92.58) | 80.82(77.45, 84.19) | 83.47(78.61, 88.34) | 84.79(81.29, 88.30) | 82.04(77.25, 86.84) | 77.04(71.59, 82.48) | 0.276(-0.563, 1.123) | 0.471 |
| **PIR** | Low income | 83.50(81.65, 85.35) | 76.03(67.35, 84.72) | 80.67(75.35, 85.98) | 80.63(76.07, 85.18) | 80.56(72.49, 88.64) | 86.08(82.54, 89.63) | 86.34(81.70, 90.98) | 87.41(84.69, 90.13) | 86.76(82.80, 90.71) | 88.00(84.13, 91.87) | 77.81(69.56, 86.06) | 0.578(0.078, 1.081) | **0.028** |
|  | Middle income | 82.89(81.49, 84.29) | 74.16(68.01, 80.31) | 78.19(73.15, 83.23) | 80.72(76.18, 85.25) | 80.60(75.94, 85.27) | 80.43(76.29, 84.57) | 83.78(80.30, 87.27) | 86.24(82.05, 90.43) | 89.72(87.36, 92.07) | 86.98(84.37, 89.58) | 83.55(79.93, 87.18) | 0.767(0.251, 1.284) | **0.009** |
|  | High income | 81.76(80.67, 82.85) | 73.04(70.23, 75.86) | 74.48(71.07, 77.89) | 80.95(76.30, 85.61) | 82.07(79.17, 84.97) | 84.45(81.77, 87.13) | 84.36(81.96, 86.77) | 82.96(79.27, 86.66) | 86.80(84.29, 89.32) | 82.56(78.52, 86.61) | 82.12(78.47, 85.77) | 0.751(0.201, 1.304) | **0.014** |
| **Health insurance** | No | 76.57(74.30, 78.84) | 74.68(66.23, 83.14) | 73.99(67.24, 80.75) | 73.20(64.82, 81.59) | 77.09(66.50, 87.69) | 68.90(63.55, 74.25) | 77.84(72.79, 82.88) | 80.38(74.38, 86.39) | 78.85(73.51, 84.19) | 82.53(77.77, 87.29) | 76.84(68.31, 85.38) | 0.707(0.062, 1.357) | **0.035** |
|  | Yes | 82.82(81.94, 83.69) | 73.14(70.14, 76.14) | 76.19(73.76, 78.63) | 81.83(78.40, 85.27) | 82.49(80.13, 84.85) | 86.20(84.59, 87.80) | 85.32(83.19, 87.45) | 85.18(82.59, 87.77) | 88.65(86.66, 90.63) | 84.20(81.33, 87.06) | 81.62(77.96, 85.28) | 0.742(0.115, 1.373) | **0.026** |
| **Life’s simple 7** | Inadequate health | 80.68(79.10, 82.25) | 70.86(65.13, 76.60) | 71.80(64.73, 78.87) | 82.44(77.09, 87.78) | 78.06(73.19, 82.92) | 80.92(76.96, 84.88) | 85.48(81.71, 89.25) | 82.66(78.97, 86.35) | 84.83(81.88, 87.77) | 83.12(77.81, 88.43) | 80.87(73.85, 87.89) | 0.751(0.133, 1.373) | **0.023** |
|  | Average health | 81.64(80.69, 82.60) | 73.35(70.16, 76.55) | 75.75(73.08, 78.43) | 79.33(75.73, 82.92) | 81.67(78.78, 84.55) | 84.14(81.55, 86.72) | 83.47(80.61, 86.33) | 84.10(80.77, 87.43) | 88.25(85.51, 90.99) | 83.70(80.86, 86.54) | 80.16(77.31, 83.02) | 0.611(0.055, 1.171) | **0.035** |
|  | Optimal health | 89.09(87.00, 91.18) | 79.41(65.52, 93.29) | 89.30(82.01, 96.58) | 89.91(83.93, 95.89) | 89.71(86.07, 93.35) | 87.57(82.73, 92.41) | 89.05(85.01, 93.09) | 90.14(83.99, 96.28) | 98.29(95.12, 101.47) | 89.08(83.04, 95.11) | 89.92(82.08, 97.76) | 0.607(-0.101, 1.32) | 0.083 |

PIR: income-to-poverty ratio; AAPC: average annual percent change

**sTable 9 Annual prevalence change of awareness among US hypertension adults from 1999 to 2018**

| **Characteristics** | | **Jointpoint** | **APC1** | ***P*** | **APC2** | ***P*** | **AAPC** | ***P*** |
| --- | --- | --- | --- | --- | --- | --- | --- | --- |
| **Total** |  | 2013 | 1.127(0.59, 1.668) | **0.003** | -2.101(-6.375, 2.368) | 0.276 | 0.737(0.241, 1.235) | **0.009** |
| **Age** | 20~ | 2013 | 1.589(0.54, 2.648) | **0.011** | -1.786(-6.55, 3.222) | 0.394 | 1.028(0.229, 1.833) | **0.018** |
|  | 40~ | 2009 | 1.229(0.727, 1.734) | **0.001** | -0.887(-1.653, -0.115) | **0.032** | 0.384(-0.104, 0.875) | 0.107 |
|  | 60~ | 2013 | 1.284(0.585, 1.989) | **0.005** | -1.921(-7.523, 4.02) | 0.435 | 0.952(0.419, 1.487) | **0.003** |
| **Sex** | Female | 2013 | 1.062(0.457, 1.669) | **0.006** | -2.587(-7.354, 2.426) | 0.237 | 0.661(0.09, 1.235) | **0.028** |
|  | Male | 2007 | 1.916(0.283, 3.575) | **0.029** | 0.024(-0.906, 0.962) | 0.951 | 0.858(0.378, 1.339) | **0.003** |
| **Race** | Non-Hispanic white | 2013 | 1.23(0.58, 1.884) | **0.005** | -2.286(-8.571, 4.431) | 0.412 | 0.881(0.352, 1.413) | **0.005** |
|  | Non-Hispanic black | 2013 | 0.736(0.204, 1.271) | **0.016** | -1.326(-4.274, 1.712) | 0.309 | 0.365(-0.037, 0.768) | 0.07 |
|  | Mexican American | 2013 | 1.268(-0.054, 2.608) | 0.057 | -3.42(-11.912, 5.891) | 0.376 | 0.639(-0.303, 1.59) | 0.157 |
|  | Other Hispanic | 2009 | 2.354(1.333, 3.386) | **0.002** | -0.857(-1.628, -0.079) | **0.037** | 0.5(-0.567, 1.578) | 0.313 |
|  | Other race | 2003 | 6.482(-3.039, 16.939) | 0.145 | 0.06(-0.514, 0.636) | 0.8 | 0.483(-0.144, 1.113) | 0.114 |
| **Marital status** | No | 2013 | 1.199(0.828, 1.571) | **<0.001** | -1.96(-4.636, 0.79) | 0.125 | 0.822(0.354, 1.292) | **0.004** |
|  | Yes | 2007 | 2.026(0.888, 3.176) | **0.006** | -0.34(-1.282, 0.611) | 0.399 | 0.682(0.123, 1.244) | **0.023** |
| **Education level** | Less than high school | 2011 | 1.311(0.977, 1.647) | **<0.001** | -0.935(-1.835, -0.026) | **0.046** | 0.605(0.137, 1.075) | **0.017** |
|  | High school graduate | 2009 | 1.571(0.598, 2.553) | **0.009** | -0.702(-2.321, 0.943) | 0.321 | 0.484(-0.11, 1.082) | 0.097 |
|  | Some college | 2013 | 1.437(0.887, 1.99) | **0.001** | -1.739(-5.521, 2.194) | 0.303 | 1.115(0.521, 1.713) | **0.002** |
|  | College graduate or above | 2007 | 2.294(-1.033, 5.732) | 0.138 | -0.641(-1.902, 0.636) | 0.252 | 0.276(-0.563, 1.123) | 0.471 |
| **PIR** | Low income | 2011 | 0.998(-0.023, 2.029) | 0.054 | -0.615(-3.6, 2.462) | 0.625 | 0.578(0.078, 1.081) | **0.028** |
|  | Middle income | 2013 | 1.247(0.808, 1.687) | **0.001** | -1.481(-3.481, 0.562) | 0.121 | 0.767(0.251, 1.284) | **0.009** |
|  | High income | 2007 | 1.937(0.573, 3.321) | **0.015** | -0.142(-1.073, 0.797) | 0.712 | 0.751(0.201, 1.304) | **0.014** |
| **Health insurance** | No | 2007 | -0.13(-2.535, 2.335) | 0.896 | 1.246(0.166, 2.338) | **0.031** | 0.707(0.062, 1.357) | **0.035** |
|  | Yes | 2007 | 2.045(0.254, 3.869) | **0.032** | -0.125(-1.322, 1.086) | 0.8 | 0.742(0.115, 1.373) | **0.026** |
| **Life’s simple 7** | Inadequate health | 2009 | 1.602(-0.06, 3.292) | 0.056 | -0.317(-2.469, 1.882) | 0.724 | 0.751(0.133, 1.373) | **0.023** |
|  | Average health | 2013 | 1.187(0.646, 1.731) | **0.002** | -2.426(-5.867, 1.141) | 0.139 | 0.611(0.055, 1.171) | **0.035** |
|  | Optimal health | 2013 | 0.93(0.269, 1.595) | **0.015** | -2.155(-6.791, 2.712) | 0.301 | 0.607(-0.101, 1.32) | 0.083 |

PIR: income-to-poverty ratio; APC1: annual rate of change before inflection; APC2: annual rate of change after inflection; AAPC: average annual rates of change

**sTable 10 Prevalence of treatment among US hypertension adults from 1999 to 2018**

|  | | | | | | | | | | | | |  |  |
| --- | --- | --- | --- | --- | --- | --- | --- | --- | --- | --- | --- | --- | --- | --- |
| **Characteristics** | | **Total (n=22947)** | **1999-2000 (n=1952)** | **2001-2002 (n=2096)** | **2003-2004 (n=2131)** | **2005-2006 (n=1903)** | **2007-2008 (n=2535)** | **2009-2010 (n=2548)** | **2011-2012 (n=2334)** | **2013-2014 (n=2451)** | **2015-2016 (n=2419)** | **2017-2018 (n=2578)** | **AAPC** | ***P*** |
| **Total** |  | 60.00(57.11, 62.89) | 51.16(46.40, 55.93) | 51.07(47.33, 54.82) | 56.22(51.89, 60.55) | 58.58(53.79, 63.36) | 62.58(60.21, 64.95) | 67.47(64.58, 70.35) | 63.38(58.54, 68.23) | 62.50(58.80, 66.20) | 62.90(60.39, 65.41) | 59.52(56.14, 62.90) | 0.764(-0.276, 1.815) | 0.129 |
| **Age (years)** | 20-49 | 22.79(20.67, 24.91) | 17.20(10.87, 23.53) | 19.04(15.29, 22.78) | 18.70(12.24, 25.15) | 19.83(12.94, 26.71) | 29.16(20.21, 38.11) | 25.70(20.17, 31.24) | 24.69(17.64, 31.74) | 25.57(20.91, 30.23) | 27.62(19.84, 35.40) | 20.07(14.22, 25.92) | 1.900(0.026, 3.808) | **0.047** |
|  | 40-59 | 56.86(55.00, 58.72) | 53.31(47.36, 59.25) | 50.56(45.27, 55.84) | 55.11(48.43, 61.79) | 54.13(49.77, 58.49) | 58.55(53.92, 63.19) | 68.03(64.25, 71.81) | 58.60(51.85, 65.34) | 56.14(48.27, 64.01) | 58.30(53.53, 63.08) | 55.18(49.35, 61.01) | 0.660(-0.820, 2.162) | 0.336 |
|  | 60-85 | 74.31(73.06, 75.57) | 62.10(55.80, 68.40) | 64.83(60.68, 68.98) | 70.92(66.20, 75.63) | 74.95(71.42, 78.48) | 76.62(74.48, 78.76) | 78.63(75.25, 82.01) | 78.80(75.53, 82.06) | 79.56(75.91, 83.21) | 76.31(72.50, 80.12) | 74.68(70.49, 78.88) | 0.774(-0.018, 1.572) | 0.054 |
| **Sex** | Female | 64.98(63.48, 66.47) | 54.70(49.52, 59.89) | 56.81(51.45, 62.18) | 58.37(53.13, 63.61) | 65.76(61.10, 70.42) | 67.46(63.88, 71.03) | 73.25(69.28, 77.22) | 69.92(64.53, 75.31) | 66.75(62.40, 71.09) | 69.56(66.39, 72.72) | 63.42(57.94, 68.91) | 0.969(-0.044, 1.991) | 0.058 |
|  | Male | 54.75(53.35, 56.15) | 47.24(41.69, 52.79) | 44.31(41.00, 47.61) | 53.88(49.56, 58.21) | 51.09(45.30, 56.88) | 57.37(53.37, 61.37) | 61.56(58.47, 64.64) | 56.71(51.18, 62.24) | 57.96(54.24, 61.68) | 56.14(52.23, 60.06) | 55.77(51.83, 59.72) | 1.083(-0.108, 2.288) | 0.069 |
| **Race** | Non-Hispanic white | 61.04(59.53, 62.55) | 53.38(48.36, 58.39) | 51.15(46.77, 55.53) | 56.33(51.94, 60.73) | 59.41(53.54, 65.29) | 64.40(61.31, 67.50) | 68.88(65.01, 72.74) | 66.10(59.89, 72.30) | 63.60(58.92, 68.28) | 64.72(60.87, 68.57) | 59.09(53.80, 64.38) | 1.032(-0.09, 2.167) | 0.067 |
|  | Non-Hispanic black | 64.15(62.58, 65.72) | 55.71(49.80, 61.62) | 55.30(51.34, 59.26) | 59.60(53.59, 65.61) | 66.85(60.90, 72.80) | 64.21(58.83, 69.58) | 69.70(63.89, 75.51) | 66.75(62.63, 70.87) | 66.53(62.36, 70.69) | 67.26(63.77, 70.76) | 63.48(58.27, 68.69) | 0.968(0.241, 1.701) | **0.015** |
|  | Mexican American | 47.14(44.24, 50.03) | 32.20(26.06, 38.34) | 32.17(25.62, 38.73) | 45.95(36.76, 55.13) | 37.19(27.04, 47.34) | 49.43(44.27, 54.58) | 53.93(44.21, 63.64) | 44.85(35.83, 53.88) | 54.69(48.86, 60.52) | 50.10(41.68, 58.53) | 53.21(46.33, 60.08) | 2.510(0.931, 4.115) | **0.006** |
|  | Other Hispanic | 51.49(48.11, 54.87) | 46.25(33.11, 59.39) | 44.91(32.25, 57.57) | 49.02(30.12, 67.92) | 37.17(21.21, 53.12) | 56.20(47.03, 65.36) | 52.87(43.52, 62.22) | 56.12(48.90, 63.33) | 51.08(43.65, 58.52) | 54.09(46.18, 62.01) | 54.88(50.00, 59.76) | 0.768(-0.196, 1.740) | 0.104 |
|  | Other race | 56.72(53.33, 60.12) | 31.69(23.44, 39.94) | 62.52(46.29, 78.75) | 60.28(44.88, 75.67) | 51.99(36.42, 67.55) | 50.08(33.65, 66.52) | 68.73(60.19, 77.28) | 48.87(36.96, 60.77) | 56.46(49.26, 63.65) | 58.43(53.49, 63.37) | 63.34(55.69, 70.98) | 1.166(-1.059, 3.441) | 0.264 |
| **Marital status** | No | 57.22(55.70, 58.75) | 45.80(38.78, 52.82) | 51.02(46.50, 55.54) | 54.42(48.43, 60.41) | 57.04(50.80, 63.27) | 56.59(54.47, 58.72) | 63.12(59.43, 66.81) | 60.09(54.41, 65.77) | 59.76(55.88, 63.63) | 61.34(58.66, 64.02) | 55.72(50.29, 61.15) | 0.985(0.167, 1.809) | **0.024** |
|  | Yes | 62.15(60.66, 63.64) | 54.56(48.91, 60.21) | 51.15(46.60, 55.70) | 57.53(52.84, 62.21) | 59.63(54.60, 64.66) | 66.64(62.72, 70.56) | 70.35(66.98, 73.72) | 66.09(59.46, 72.71) | 64.52(59.49, 69.54) | 64.26(60.42, 68.09) | 62.47(59.01, 65.93) | 0.767(-0.402, 1.950) | 0.169 |
| **Education level** | Less than high school | 61.80(59.90, 63.70) | 56.96(49.63, 64.29) | 54.52(49.82, 59.22) | 58.41(51.88, 64.94) | 58.94(53.76, 64.11) | 63.04(58.39, 67.69) | 69.40(65.57, 73.24) | 64.33(56.08, 72.59) | 67.48(62.12, 72.85) | 62.19(56.86, 67.52) | 62.59(55.43, 69.75) | 0.992(-0.043, 2.037) | 0.058 |
|  | High school graduate | 60.67(58.76, 62.57) | 49.32(41.79, 56.86) | 50.45(44.46, 56.44) | 55.68(51.44, 59.91) | 63.62(57.13, 70.11) | 66.73(62.46, 71.01) | 69.83(65.09, 74.57) | 60.50(54.52, 66.49) | 62.40(54.59, 70.20) | 65.30(60.79, 69.81) | 60.12(54.08, 66.15) | 0.983(-0.389, 2.373) | 0.138 |
|  | Some college | 59.46(57.56, 61.37) | 50.03(46.14, 53.91) | 52.64(45.74, 59.53) | 55.73(47.89, 63.57) | 55.93(49.34, 62.52) | 57.01(50.50, 63.52) | 64.70(60.76, 68.64) | 61.05(52.83, 69.27) | 63.67(58.37, 68.97) | 62.78(58.36, 67.20) | 61.70(57.23, 66.16) | 1.219(0.523, 1.919) | **0.004** |
|  | College graduate or above | 58.39(56.34, 60.43) | 46.70(36.61, 56.79) | 45.77(41.30, 50.23) | 55.38(47.36, 63.41) | 55.52(48.40, 62.64) | 64.02(57.74, 70.30) | 67.11(60.94, 73.27) | 67.92(62.37, 73.47) | 57.54(52.01, 63.07) | 61.22(56.51, 65.94) | 54.69(48.49, 60.88) | 1.198(-0.530, 2.956) | 0.149 |
| **PIR** | Low income | 55.43(53.06, 57.80) | 49.84(42.31, 57.38) | 51.04(43.05, 59.03) | 53.21(48.34, 58.07) | 57.92(50.00, 65.84) | 56.79(50.84, 62.73) | 55.88(48.22, 63.55) | 56.71(48.91, 64.51) | 56.61(49.32, 63.89) | 63.20(57.23, 69.17) | 48.79(41.49, 56.10) | 0.704(-0.262, 1.680) | 0.132 |
|  | Middle income | 61.19(59.14, 63.24) | 49.23(40.09, 58.37) | 53.66(47.06, 60.27) | 58.48(50.82, 66.14) | 60.78(55.69, 65.88) | 65.81(61.82, 69.81) | 67.83(63.69, 71.97) | 63.46(56.24, 70.68) | 60.82(54.59, 67.04) | 64.09(58.43, 69.74) | 63.52(57.89, 69.14) | 0.695(-0.390, 1.792) | 0.179 |
|  | High income | 60.55(59.13, 61.97) | 52.17(47.20, 57.15) | 49.73(44.79, 54.67) | 56.03(51.93, 60.12) | 57.66(51.28, 64.04) | 63.69(60.79, 66.58) | 70.38(66.68, 74.08) | 64.17(59.18, 69.15) | 64.05(59.38, 68.73) | 62.55(59.49, 65.60) | 60.64(56.91, 64.38) | 0.789(-0.410, 2.001) | 0.168 |
| **Health insurance** | No | 34.56(32.17, 36.94) | 31.86(18.54, 45.17) | 23.38(18.55, 28.21) | 31.61(23.87, 39.36) | 30.17(23.57, 36.76) | 35.33(29.70, 40.95) | 42.92(38.44, 47.41) | 33.97(25.27, 42.67) | 36.97(29.55, 44.39) | 39.89(32.32, 47.46) | 34.67(28.68, 40.66) | 1.872(-0.637, 4.445) | 0.125 |
|  | Yes | 63.42(62.17, 64.67) | 53.77(48.80, 58.73) | 54.38(50.18, 58.57) | 59.73(55.75, 63.72) | 62.74(57.87, 67.62) | 66.65(63.85, 69.45) | 71.27(68.61, 73.94) | 67.93(62.80, 73.06) | 65.93(62.09, 69.77) | 65.37(62.47, 68.27) | 62.27(58.37, 66.17) | 0.710(-0.440, 1.873) | 0.193 |
| **Life’s simple 7** | Inadequate health | 63.96(61.78, 66.15) | 54.33(44.73, 63.92) | 54.55(47.81, 61.30) | 63.23(57.08, 69.38) | 60.82(54.54, 67.09) | 67.22(63.06, 71.38) | 75.26(69.45, 81.07) | 61.83(55.29, 68.36) | 67.39(61.41, 73.37) | 64.42(56.85, 71.99) | 65.26(57.47, 73.05) | 0.871(-0.227, 1.981) | 0.105 |
|  | Average health | 59.95(58.63, 61.27) | 50.96(45.78, 56.14) | 51.32(47.45, 55.18) | 55.74(51.06, 60.41) | 58.67(52.66, 64.68) | 61.95(59.55, 64.36) | 66.64(63.84, 69.45) | 65.20(60.27, 70.13) | 61.29(58.08, 64.51) | 64.17(60.12, 68.23) | 59.33(55.60, 63.06) | 0.820(-0.535, 2.193) | 0.202 |
|  | Optimal health | 51.47(47.57, 55.36) | 43.21(28.34, 58.07) | 37.48(26.39, 48.58) | 45.28(31.58, 58.98) | 53.29(42.45, 64.12) | 59.34(51.61, 67.07) | 61.12(52.03, 70.21) | 53.05(42.33, 63.77) | 39.05(25.18, 52.93) | 49.56(38.35, 60.76) | 51.13(37.72, 64.55) | 0.567(-1.993, 3.193) | 0.627 |

PIR: income-to-poverty ratio; AAPC: average annual percent change

**sTable 11 Annual prevalence change of treatment among US hypertension adults from 1999 to 2018**

| **Characteristics** | | **Jointpoint** | **APC1** | ***P*** | **APC2** | ***P*** | **AAPC** | ***P*** |
| --- | --- | --- | --- | --- | --- | --- | --- | --- |
| **Total** |  | 2009 | 3.053(2.136, 3.978) | **<0.001** | -1.242(-2.532, 0.066) | 0.058 | 0.764(-0.276, 1.815) | 0.129 |
| **Age** | 20~ | 2007 | 6.196(-0.795, 13.679) | 0.073 | -1.243(-4.721, 2.361) | 0.411 | 1.900(0.026, 3.808) | **0.047** |
|  | 40~ | 2009 | 2.837(1.418, 4.275) | **0.004** | -2.039(-4.269, 0.243) | 0.070 | 0.660(-0.820, 2.162) | 0.336 |
|  | 60~ | 2009 | 2.378(1.517, 3.246) | **0.001** | -0.764(-1.805, 0.288) | 0.121 | 0.774(-0.018, 1.572) | 0.054 |
| **Sex** | Female | 2009 | 3.013(1.487, 4.562) | **0.004** | -1.114(-3.244, 1.063) | 0.243 | 0.969(-0.044, 1.991) | 0.058 |
|  | Male | 2009 | 3.219(0.930, 5.559) | **0.015** | -1.242(-4.094, 1.694) | 0.323 | 1.083(-0.108, 2.288) | 0.069 |
| **Race** | Non-Hispanic white | 2009 | 3.101(1.713, 4.507) | **0.002** | -1.373(-3.718, 1.029) | 0.200 | 1.032(-0.09, 2.167) | 0.067 |
|  | Non-Hispanic black | 2009 | 2.413(0.677, 4.180) | **0.016** | -0.737(-2.553, 1.114) | 0.351 | 0.968(0.241, 1.701) | **0.015** |
|  | Mexican American | 2007 | 5.824(-2.512, 14.872) | 0.136 | 0.735(-3.294, 4.931) | 0.664 | 2.510(0.931, 4.115) | **0.006** |
|  | Other Hispanic | 2007 | 2.590(-3.422, 8.975) | 0.326 | 0.117(-1.381, 1.637) | 0.850 | 0.768(-0.196, 1.740) | 0.104 |
|  | Other race | 2003 | 14.362(-20.474, 64.458) | 0.386 | -0.353(-3.507, 2.905) | 0.789 | 1.166(-1.059, 3.441) | 0.264 |
| **Marital status** | No | 2009 | 2.540(0.957, 4.147) | **0.009** | -0.277(-2.684, 2.190) | 0.782 | 0.985(0.167, 1.809) | **0.024** |
|  | Yes | 2009 | 3.227(1.662, 4.817) | **0.003** | -1.432(-3.432, 0.609) | 0.130 | 0.767(-0.402, 1.950) | 0.169 |
| **Education level** | Less than high school | 2009 | 2.584(1.143, 4.045) | **0.006** | -1.066(-3.455, 1.383) | 0.311 | 0.992(-0.043, 2.037) | 0.058 |
|  | High school graduate | 2007 | 4.655(0.494, 8.989) | **0.034** | -0.885(-2.718, 0.982) | 0.275 | 0.983(-0.389, 2.373) | 0.138 |
|  | Some college | 2009 | 2.482(1.700, 3.270) | **<0.001** | -0.290(-1.334, 0.766) | 0.510 | 1.219(0.523, 1.919) | **0.004** |
|  | College graduate or above | 2009 | 4.732(1.711, 7.843) | **0.010** | -2.512(-5.453, 0.520) | 0.086 | 1.198(-0.530, 2.956) | 0.149 |
| **PIR** | Low income | 2013 | 1.282(0.409, 2.162) | **0.013** | -2.459(-8.003, 3.418) | 0.324 | 0.704(-0.262, 1.680) | 0.132 |
|  | Middle income | 2007 | 3.724(1.214, 6.296) | **0.012** | -0.557(-1.609, 0.507) | 0.235 | 0.695(-0.390, 1.792) | 0.179 |
|  | High income | 2009 | 3.370(2.208, 4.546) | **0.001** | -1.548(-3.071, -0.002) | 0.050 | 0.789(-0.410, 2.001) | 0.168 |
| **Health insurance** | No | 2009 | 6.060(0.935, 11.445) | **0.028** | -1.894(-7.889, 4.492) | 0.471 | 1.872(-0.637, 4.445) | 0.125 |
|  | Yes | 2009 | 3.049(2.396, 3.705) | **<0.001** | -1.528(-2.441, -0.606) | **0.008** | 0.710(-0.440, 1.873) | 0.193 |
| **Life’s simple 7** | Inadequate health | 2009 | 2.965(1.799, 4.143) | **0.001** | -1.179(-2.804, 0.473) | 0.125 | 0.871(-0.227, 1.981) | 0.105 |
|  | Average health | 2009 | 2.941(1.084, 4.832) | **0.009** | -1.579(-4.238, 1.153) | 0.195 | 0.820(-0.535, 2.193) | 0.202 |
|  | Optimal health | 2007 | 6.064(-1.821, 14.582) | **0.107** | -2.299(-6.235, 1.801) | 0.206 | 0.567(-1.993, 3.193) | 0.627 |

PIR: income-to-poverty ratio; APC1: annual rate of change before inflection; APC2: annual rate of change after inflection; AAPC: average annual rates of change

**sTable 12 Prevalence of control among US hypertension adults from 1999 to 2018**

| **Characteristics** | | **Total (n=22947)** | **1999-2000 (n=1952)** | **2001-2002 (n=2096)** | **2003-2004 (n=2131)** | **2005-2006 (n=1903)** | **2007-2008 (n=2535)** | **2009-2010 (n=2548)** | **2011-2012 (n=2334)** | **2013-2014 (n=2451)** | **2015-2016 (n=2419)** | **2017-2018 (n=2578)** | **AAPC** | ***P*** |
| --- | --- | --- | --- | --- | --- | --- | --- | --- | --- | --- | --- | --- | --- | --- |
| **Total** |  | 52.46(49.87, 55.05) | 41.20(37.24, 45.17) | 46.13(44.46, 47.81) | 51.26(46.62, 55.90) | 53.71(50.72, 56.71) | 58.05(55.13, 60.96) | 60.05(57.45, 62.64) | 59.03(55.56, 62.51) | 62.91(58.26, 67.55) | 55.89(51.96, 59.83) | 51.71(48.54, 54.88) | 1.475(0.156, 2.811) | **0.033** |
| **Age (years)** | 20-49 | 61.14(58.59, 63.68) | 44.14(34.14, 54.14) | 60.19(54.43, 65.96) | 62.58(50.87, 74.30) | 58.62(52.41, 64.82) | 66.12(59.65, 72.59) | 59.60(53.74, 65.46) | 61.80(54.67, 68.92) | 70.19(63.74, 76.65) | 63.19(56.87, 69.51) | 60.96(54.54, 67.39) | 0.663(-0.417, 1.754) | 0.196 |
|  | 40-59 | 57.31(55.51, 59.12) | 50.95(45.16, 56.74) | 48.36(45.04, 51.68) | 52.72(45.23, 60.21) | 57.16(52.72, 61.61) | 60.06(54.49, 65.64) | 61.87(57.97, 65.78) | 64.80(58.94, 70.66) | 66.06(59.62, 72.51) | 56.85(51.33, 62.37) | 51.40(46.23, 56.57) | 1.056(-0.317, 2.449) | 0.114 |
|  | 60-85 | 49.79(48.38, 51.19) | 31.91(28.14, 35.68) | 38.18(35.27, 41.09) | 45.70(41.47, 49.92) | 48.88(44.50, 53.27) | 53.69(49.68, 57.70) | 58.75(55.22, 62.28) | 53.21(49.22, 57.19) | 57.97(54.07, 61.86) | 53.15(47.84, 58.46) | 49.14(44.12, 54.16) | 2.445(0.579, 4.345) | **0.016** |
| **Sex** | Female | 55.11(53.83, 56.39) | 40.53(35.86, 45.20) | 45.91(42.23, 49.59) | 50.01(46.17, 53.84) | 54.79(51.25, 58.34) | 59.51(57.09, 61.94) | 62.60(58.65, 66.55) | 60.98(56.84, 65.12) | 65.99(61.67, 70.31) | 57.57(53.04, 62.09) | 50.32(47.19, 53.45) | 0.992(-0.791, 2.806) | 0.237 |
|  | Male | 53.61(51.96, 55.25) | 41.94(36.72, 47.16) | 46.39(43.57, 49.22) | 52.57(45.48, 59.66) | 52.60(48.19, 57.01) | 56.47(51.88, 61.05) | 57.48(54.64, 60.31) | 57.07(53.51, 60.64) | 59.71(53.49, 65.92) | 54.21(48.13, 60.28) | 53.01(47.90, 58.12) | 1.409(0.346, 2.484) | **0.016** |
| **Race** | Non-Hispanic white | 55.75(54.29, 57.21) | 42.69(38.65, 46.73) | 47.06(45.18, 48.95) | 52.72(47.43, 58.01) | 55.22(52.12, 58.33) | 59.70(56.72, 62.68) | 61.91(58.63, 65.19) | 59.60(55.35, 63.85) | 65.61(59.31, 71.91) | 56.63(51.27, 62.00) | 53.37(48.92, 57.82) | 1.729(0.373, 3.104) | **0.019** |
|  | Non-Hispanic black | 49.65(48.15, 51.14) | 38.45(30.48, 46.43) | 43.33(40.29, 46.36) | 48.01(42.30, 53.71) | 48.91(44.31, 53.52) | 51.57(47.51, 55.64) | 55.15(50.13, 60.18) | 54.91(50.57, 59.25) | 54.76(49.99, 59.53) | 51.88(48.26, 55.50) | 45.15(41.68, 48.61) | 0.726(-0.52, 1.988) | 0.217 |
|  | Mexican American | 50.98(48.48, 53.47) | 35.37(27.26, 43.48) | 38.93(31.84, 46.02) | 49.07(42.39, 55.76) | 50.57(42.45, 58.68) | 54.88(49.40, 60.36) | 48.20(45.26, 51.15) | 60.89(49.04, 72.73) | 57.29(50.62, 63.95) | 57.81(52.70, 62.92) | 44.60(36.84, 52.37) | 1.635(-0.131, 3.431) | 0.065 |
|  | Other Hispanic | 52.91(48.73, 57.09) | 40.80(24.14, 57.46) | 49.91(37.03, 62.79) | 49.17(34.21, 64.12) | 37.07(24.07, 50.07) | 55.73(49.75, 61.72) | 64.00(54.77, 73.22) | 61.24(55.15, 67.33) | 64.73(57.37, 72.09) | 58.74(52.21, 65.28) | 44.43(34.02, 54.85) | 1.152(-1.061, 3.415) | 0.267 |
|  | Other race | 53.11(50.01, 56.21) | 32.00(16.95, 47.06) | 39.87(21.98, 57.76) | 39.31(31.63, 46.99) | 54.29(43.14, 65.45) | 56.61(46.48, 66.74) | 59.21(48.94, 69.48) | 58.76(50.16, 67.37) | 54.93(47.69, 62.17) | 53.24(45.34, 61.14) | 58.45(53.59, 63.31) | 1.59(0.074, 3.129) | **0.042** |
| **Marital status** | No | 52.47(50.97, 53.97) | 37.49(33.47, 41.51) | 45.26(41.66, 48.86) | 47.95(44.27, 51.63) | 51.49(46.68, 56.31) | 54.77(52.23, 57.31) | 57.28(54.10, 60.46) | 57.46(52.19, 62.72) | 63.21(57.91, 68.51) | 53.49(49.56, 57.42) | 49.40(44.57, 54.23) | 1.523(0.015, 3.055) | **0.048** |
|  | Yes | 55.94(54.50, 57.39) | 43.37(38.32, 48.42) | 46.80(44.42, 49.17) | 53.23(47.54, 58.92) | 55.36(50.82, 59.91) | 60.27(56.23, 64.32) | 61.84(58.63, 65.06) | 60.33(56.69, 63.98) | 62.70(56.88, 68.53) | 57.96(53.27, 62.65) | 53.48(49.64, 57.32) | 1.378(0.048, 2.726) | **0.044** |
| **Education level** | Less than high school | 49.67(47.97, 51.37) | 34.09(29.31, 38.88) | 42.51(37.29, 47.73) | 47.78(43.11, 52.44) | 46.86(41.84, 51.89) | 53.36(48.95, 57.76) | 58.11(54.79, 61.43) | 58.11(53.05, 63.17) | 61.49(54.98, 67.99) | 52.60(46.46, 58.74) | 42.45(37.10, 47.79) | 1.727(-0.51, 4.014) | 0.114 |
|  | High school graduate | 52.86(50.91, 54.80) | 40.83(33.26, 48.41) | 47.49(43.10, 51.87) | 51.10(44.32, 57.89) | 52.00(46.21, 57.79) | 55.68(52.67, 58.70) | 61.24(55.52, 66.96) | 55.15(50.16, 60.14) | 58.68(52.15, 65.21) | 55.94(49.29, 62.60) | 50.44(43.16, 57.73) | 1.129(-0.115, 2.389) | 0.07 |
|  | Some college | 56.20(54.34, 58.05) | 44.34(37.29, 51.39) | 47.56(41.64, 53.49) | 50.66(44.16, 57.16) | 54.97(49.07, 60.88) | 59.79(55.02, 64.55) | 59.28(53.36, 65.20) | 62.22(57.42, 67.01) | 66.58(61.77, 71.38) | 56.79(49.97, 63.60) | 52.26(48.09, 56.43) | 0.949(-0.642, 2.566) | 0.207 |
|  | College graduate or above | 57.97(55.75, 60.20) | 49.72(38.31, 61.12) | 46.85(41.78, 51.92) | 56.72(47.73, 65.71) | 60.14(53.75, 66.53) | 63.94(57.22, 70.65) | 62.44(57.83, 67.05) | 59.12(51.48, 66.76) | 63.02(56.14, 69.89) | 56.76(49.22, 64.31) | 56.73(51.86, 61.60) | 0.642(-0.681, 1.984) | 0.297 |
| **PIR** | Low income | 53.06(50.60, 55.52) | 40.47(30.37, 50.56) | 47.88(41.90, 53.86) | 47.19(40.99, 53.39) | 46.15(37.36, 54.93) | 59.65(52.09, 67.21) | 58.80(53.50, 64.09) | 57.70(52.38, 63.03) | 61.69(53.68, 69.70) | 57.58(52.58, 62.58) | 46.02(39.12, 52.92) | 1.139(-0.434, 2.738) | 0.134 |
|  | Middle income | 51.16(49.10, 53.21) | 38.57(31.92, 45.22) | 42.63(35.56, 49.71) | 49.18(43.11, 55.26) | 46.11(41.36, 50.86) | 47.73(42.65, 52.81) | 55.25(50.35, 60.14) | 59.43(54.86, 64.00) | 63.40(56.52, 70.29) | 56.44(50.35, 62.53) | 46.05(40.11, 51.99) | 1.798(0.012, 3.616) | **0.049** |
|  | High income | 56.13(54.68, 57.58) | 43.86(39.23, 48.48) | 47.44(44.40, 50.49) | 52.73(46.19, 59.26) | 56.59(52.39, 60.79) | 60.49(56.52, 64.46) | 62.57(59.76, 65.37) | 59.26(55.04, 63.48) | 62.84(58.41, 67.26) | 57.18(51.19, 63.17) | 54.46(50.68, 58.24) | 1.235(-0.17, 2.66) | 0.077 |
| **Health insurance** | No | 48.93(46.12, 51.74) | 38.39(28.24, 48.54) | 46.44(39.00, 53.88) | 44.06(35.09, 53.04) | 53.91(42.34, 65.48) | 46.27(38.43, 54.11) | 47.94(42.80, 53.08) | 58.25(49.26, 67.24) | 56.83(49.39, 64.26) | 52.38(46.33, 58.44) | 39.21(31.05, 47.37) | 0.94(-0.775, 2.684) | 0.243 |
|  | Yes | 55.16(53.96, 56.36) | 42.03(38.21, 45.85) | 46.11(44.36, 47.87) | 52.32(47.85, 56.78) | 53.76(50.47, 57.06) | 59.84(56.89, 62.79) | 61.91(59.34, 64.48) | 59.14(55.53, 62.75) | 63.71(59.16, 68.26) | 56.24(51.89, 60.59) | 53.08(49.52, 56.63) | 1.686(0.262, 3.13) | **0.026** |
| **Life’s simple 7** | Inadequate health | 36.79(34.80, 38.78) | 23.66(17.06, 30.26) | 23.75(19.01, 28.50) | 33.23(26.37, 40.09) | 31.71(27.36, 36.07) | 34.94(29.95, 39.92) | 43.22(36.76, 49.67) | 41.54(33.99, 49.08) | 50.04(44.70, 55.39) | 37.96(28.68, 47.24) | 32.33(27.65, 37.02) | 2.692(-0.241, 5.712) | 0.067 |
|  | Average health | 55.47(54.12, 56.82) | 42.73(38.31, 47.15) | 48.61(46.82, 50.39) | 51.87(47.13, 56.61) | 54.91(51.37, 58.45) | 59.57(55.27, 63.86) | 60.15(56.31, 63.99) | 59.48(55.05, 63.91) | 66.82(62.06, 71.58) | 56.65(52.31, 60.99) | 51.97(48.49, 55.45) | 1.27(0.076, 2.477) | **0.04** |
|  | Optimal health | 81.64(79.21, 84.08) | 74.97(60.00, 89.94) | 74.69(65.81, 83.56) | 81.17(72.98, 89.36) | 82.83(75.86, 89.79) | 82.81(77.68, 87.95) | 85.47(81.67, 89.27) | 80.09(73.75, 86.43) | 94.20(88.11, 100.29) | 82.08(75.45, 88.71) | 81.64(72.42, 90.87) | 0.645(-0.329, 1.629) | 0.166 |

PIR: income-to-poverty ratio; AAPC: average annual percent change

**sTable 13 Annual prevalence change of control among US hypertension adults from 1999 to 2018**

| **Characteristics** | | **Jointpoint** | **APC1** | ***P*** | **APC2** | ***P*** | **AAPC** | ***P*** |
| --- | --- | --- | --- | --- | --- | --- | --- | --- |
| **Total** |  | 2009 | 3.69(2.257, 5.142) | **0.001** | -1.753(-3.805, 0.342) | 0.084 | 1.475(0.156, 2.811) | **0.033** |
| **Age** | 20~ | 2013 | 1.334(-0.831, 3.546) | 0.176 | -2.533(-14.49, 11.096) | 0.636 | 0.663(-0.417, 1.754) | 0.196 |
|  | 40~ | 2013 | 2.442(1.582, 3.308) | **0.001** | -6.985(-13.451, -0.035) | **0.049** | 1.056(-0.317, 2.449) | 0.114 |
|  | 60~ | 2009 | 5.595(3.043, 8.21) | **0.002** | -1.911(-5.218, 1.512) | 0.208 | 2.445(0.579, 4.345) | **0.016** |
| **Sex** | Female | 2013 | 3.077(1.619, 4.555) | **0.003** | -7.538(-15.886, 1.638) | 0.086 | 0.992(-0.791, 2.806) | 0.237 |
|  | Male | 2007 | 3.835(1.686, 6.029) | **0.006** | -0.572(-1.725, 0.595) | 0.262 | 1.409(0.346, 2.484) | **0.016** |
| **Race** | Non-Hispanic white | 2009 | 3.768(2.391, 5.164) | **0.001** | -1.794(-4.266, 0.742) | 0.128 | 1.729(0.373, 3.104) | **0.019** |
|  | Non-Hispanic black | 2013 | 2.257(1.484, 3.035) | **0.001** | -5.79(-9.8, -1.602) | **0.017** | 0.726(-0.52, 1.988) | 0.217 |
|  | Mexican American | 2003 | 9.366(-24.485, 58.392) | 0.562 | 0.92(-1.992, 3.919) | 0.458 | 1.635(-0.131, 3.431) | 0.065 |
|  | Other Hispanic | 2013 | 3.03(0.278, 5.856) | **0.036** | -7.634(-22.791, 10.497) | 0.306 | 1.152(-1.061, 3.415) | 0.267 |
|  | Other race | 2007 | 7.959(-0.072, 16.637) | 0.051 | -0.098(-2.108, 1.953) | 0.906 | 1.59(0.074, 3.129) | **0.042** |
| **Marital status** | No | 2013 | 3.079(1.873, 4.299) | **0.001** | -7.042(-15.18, 1.876) | 0.096 | 1.523(0.015, 3.055) | **0.048** |
|  | Yes | 2009 | 3.7(2.418, 4.997) | **0.001** | -1.755(-3.267, -0.22) | **0.032** | 1.378(0.048, 2.726) | **0.044** |
| **Education level** | Less than high school | 2013 | 3.732(2.184, 5.303) | **0.002** | -10.126(-21.479, 2.869) | 0.098 | 1.727(-0.51, 4.014) | 0.114 |
|  | High school graduate | 2009 | 3.133(1.565, 4.726) | **0.004** | -1.544(-4.11, 1.091) | 0.19 | 1.129(-0.115, 2.389) | 0.07 |
|  | Some college | 2013 | 2.655(1.741, 3.578) | **0.001** | -6.077(-11.705, -0.09) | **0.048** | 0.949(-0.642, 2.566) | 0.207 |
|  | College graduate or above | 2007 | 4.536(1.027, 8.166) | **0.021** | -1.229(-2.64, 0.202) | 0.078 | 0.642(-0.681, 1.984) | 0.297 |
| **PIR** | Low income | 2013 | 2.686(0.965, 4.436) | **0.01** | -6.572(-17.142, 5.347) | 0.205 | 1.139(-0.434, 2.738) | 0.134 |
|  | Middle income | 2013 | 3.488(2.044, 4.953) | **0.002** | -7.016(-16.402, 3.424) | 0.139 | 1.798(0.012, 3.616) | **0.049** |
|  | High income | 2009 | 3.573(2.102, 5.065) | **0.001** | -1.677(-3.488, 0.167) | 0.066 | 1.235(-0.17, 2.66) | 0.077 |
| **Health insurance** | No | 2013 | 2.301(-0.026, 4.682) | 0.052 | -6.682(-21.041, 10.288) | 0.336 | 0.94(-0.775, 2.684) | 0.243 |
|  | Yes | 2009 | 3.939(2.422, 5.479) | **0.001** | -1.826(-4.117, 0.519) | 0.101 | 1.686(0.262, 3.13) | **0.026** |
| **Life’s simple 7** | Inadequate health | 2013 | 5.551(3.12, 8.04) | **0.002** | -10.412(-24.657, 6.527) | 0.164 | 2.692(-0.241, 5.712) | 0.067 |
|  | Average health | 2013 | 2.609(1.66, 3.566) | **0.001** | -6.128(-12.992, 1.278) | 0.085 | 1.27(0.076, 2.477) | **0.04** |
|  | Optimal health | 2013 | 1.283(0.117, 2.463) | **0.037** | -2.987(-10.758, 5.46) | 0.393 | 0.645(-0.329, 1.629) | 0.166 |

PIR: income-to-poverty ratio; APC1: annual rate of change before inflection; APC2: annual rate of change after inflection; AAPC: average annual rates of change

**sTable** **14 Prevalence of antihypertensive use among US hypertension adults from 1999 to 2018**

| **Year** | **Total (n=3295)** | **1999-2000 (n=208)** | **2001-2002 (n=267)** | **2003-2004 (n=320)** | **2005-2006 (n=268)** | **2007-2008 (n=435)** | **2009-2010 (n=444)** | **2011-2012 (n=360)** | **2013-2014 (n=359)** | **2015-2016 (n=341)** | **2017-2018 (n=293)** | **AAPC** | ***P*** |
| --- | --- | --- | --- | --- | --- | --- | --- | --- | --- | --- | --- | --- | --- |
| ACEI | 35.36(32.41, 38.32) | 24.02(16.06, 31.98) | 24.13(18.17, 30.08) | 30.58(23.72, 37.45) | 36.07(30.10, 42.05) | 30.31(24.57, 36.06) | 35.14(29.14, 41.13) | 38.40(33.35, 43.46) | 44.35(38.99, 49.71) | 39.23(32.42, 46.03) | 45.71(36.25, 55.17) | 3.368(1.907, 4.849) | **0.001** |
| ARB | 33.21(29.71, 36.70) | 20.22(13.45, 26.99) | 22.52(16.11, 28.93) | 30.46(21.11, 39.81) | 33.74(24.51, 42.96) | 33.24(28.76, 37.73) | 37.84(30.75, 44.93) | 34.05(27.70, 40.40) | 33.92(27.12, 40.72) | 40.38(33.58, 47.19) | 38.38(30.94, 45.82) | 2.746(1.166, 4.35) | **0.004** |
| CCB | 16.32(14.32, 18.33) | 6.46(0.38, 12.53) | 7.44(2.66, 12.22) | 20.07(13.30, 26.85) | 23.70(15.89, 31.51) | 19.75(14.29, 25.21) | 20.72(12.58, 28.86) | 17.39(12.47, 22.31) | 19.03(11.86, 26.21) | 12.16(7.69, 16.62) | 9.05(4.89, 13.21) | -2.155(-7.287, 3.26) | 0.378 |
| β-blocker | 8.78(7.33, 10.23) | 12.71(5.37, 20.04) | 20.35(13.82, 26.88) | 9.82(6.10, 13.53) | 5.18(2.72, 7.63) | 9.99(7.04, 12.93) | 5.92(3.54, 8.30) | 9.51(5.01, 14.00) | 7.19(4.51, 9.87) | 6.65(2.95, 10.35) | 4.21(2.61, 5.81) | -6.463(-10.543, -2.197) | **0.009** |
| Diuretic | 84.97(78.43, 91.51) | 93.54(87.47, 99.62) | 92.76(88.28, 97.24) | 81.04(75.05, 87.02) | 81.18(73.52, 88.84) | 82.56(77.45, 87.67) | 78.67(69.62, 87.72) | 83.94(79.07, 88.81) | 81.71(75.16, 88.26) | 89.77(86.97, 92.57) | 91.89(88.86, 94.92) | 0.107(-0.542, 0.761) | 0.714 |

ACEI: angiotensin-converting enzyme inhibitors; ARB: angiotensin receptor blockers; CCB: calcium channel blocker; AAPC: average annual rates of change

**sTable 15 Annual prevalence change of antihypertensive use among US hypertension adults from 1999 to 2018**

| **Year** | **Jointpoint** | **APC1** | ***P*** | **APC2** | ***P*** | **AAPC** | ***P*** |
| --- | --- | --- | --- | --- | --- | --- | --- |
| ACEI | 2005 | 6.434(-5.996, 20.507) | 0.253 | 2.629(-0.251, 5.591) | 0.066 | 3.368(1.907, 4.849) | **0.001** |
| ARB | 2005 | 8.802(-2.252, 21.105) | 0.099 | 1.332(-0.536, 3.234) | 0.127 | 2.746(1.166, 4.35) | **0.004** |
| CCB | 2005 | 24.884(-5.213, 64.538) | 0.093 | -6.665(-11.516, -1.548) | **0.021** | -2.155(-7.287, 3.26) | 0.378 |
| β-blocker | 2005 | -13.136(-37.584, 20.889) | 0.323 | -4.012(-11.992, 4.692) | 0.279 | -6.463(-10.543, -2.197) | **0.009** |
| Diuretic | 2005 | -3.208(-6.864, 0.591) | 0.081 | 1.231(0.259, 2.211) | **0.022** | 0.107(-0.542, 0.761) | 0.714 |

ACEI: angiotensin-converting enzyme inhibitors; ARB: angiotensin receptor blockers; CCB: calcium channel blocker; APC1: annual rate of change before inflection; APC2: annual rate of change after inflection; AAPC: average annual rates of change
