# Supplementary material for: Trends and influence factors in the prevalence, awareness, treatment, and control of hypertension among US adults from 1999 to 2018
Source: PLoS One. 2023 Sep 28;18(9):e0292159. doi: 10.1371/journal.pone.0292159 (PMC10538741; doi:10.1371/journal.pone.0292159)
Supplement: Attachment — Submitted filename: Response to Reviewers.docx [file pone.0292159.s002.docx]

Response Letter

Dear editor

Thank you very much for your letter and advice. First, we would like to express our sincere thanks to the reviewers for the constructive and positive comments. We have revised the manuscript, and would like to re-submit it for your consideration. We have addressed the comments raised by the reviewers, and the amendments are highlighted in the revised manuscript. Point by point responses to the reviewers’ comments are listed below this letter. We hope that the revised version of the manuscript is now acceptable for publication in your journal. I look forward to hearing from you soon.

With best wishes

**Journal Requirements:**

1. Please ensure that your manuscript meets PLOS ONE's style requirements, including those for file naming. The PLOS ONE style templates can be found at 
https://journals.plos.org/plosone/s/file?id=wjVg/PLOSOne_formatting_sample_main_body.pdf and 
https://journals.plos.org/plosone/s/file?id=ba62/PLOSOne_formatting_sample_title_authors_affiliations.pdf

**Response 1**: We have ensured that our manuscript meets PLOS ONE's style requirements.

2. We note that the grant information you provided in the ‘Funding Information’ and ‘Financial Disclosure’ sections do not match.

When you resubmit, please ensure that you provide the correct grant numbers for the awards you received for your study in the ‘Funding Information’ section.

**Response 2**: We have checked the grant numbers in the revised paper.

3. PLOS requires an ORCID iD for the corresponding author in Editorial Manager on papers submitted after December 6th, 2016. Please ensure that you have an ORCID iD and that it is validated in Editorial Manager. To do this, go to ‘Update my Information’ (in the upper left-hand corner of the main menu), and click on the Fetch/Validate link next to the ORCID field. This will take you to the ORCID site and allow you to create a new iD or authenticate a pre-existing iD in Editorial Manager. Please see the following video for instructions on linking an ORCID iD to your Editorial Manager account: <https://www.youtube.com/watch?v=_xcclfuvtxQ>

**Response 3**: ORCD for the corresponding author (yang jian) ：<https://orcid.org/0000-0002-1391-1482>

4. Please include captions for your Supporting Information files at the end of your manuscript, and update any in-text citations to match accordingly. Please see our Supporting Information guidelines for more information: http://journals.plos.org/plosone/s/supporting-information.

**Response 4**: We have added Supporting Information files at the end of manuscript, and update any in-text citations to match accordingly.

5. Please review your reference list to ensure that it is complete and correct. If you have cited papers that have been retracted, please include the rationale for doing so in the manuscript text, or remove these references and replace them with relevant current references. Any changes to the reference list should be mentioned in the rebuttal letter that accompanies your revised manuscript. If you need to cite a retracted article, indicate the article’s retracted status in the References list and also include a citation and full reference for the retraction notice.

**Response 5**: We have reviewed reference list to ensure that it is complete and correct.

**Reviewer #1:**

Regarding this statement in page 4
However, there is no estimation of the trends in the prevalence, awareness, treatment, and control of hypertension over a 20-years period in the US, and few studies have explored the factors influencing hypertension incidence, awareness, treatment, and control simultaneously.
There are 2 other articles that cover the same population over an almost similar period of time
1) Trends in the Prevalence, Awareness, Treatment, and Control of Hypertension among Young Adults in the United States, 1999–2014
Yiyi Zhang, and Andrew E Moran, MD. Hypertension. 2017 Oct; 70(4): 736–742.
2)Trends in Blood Pressure Control Among US Adults With Hypertension, 1999-2000 to 2017-2018
Paul Muntner, Shakia T Hardy, Lawrence J Fine , Byron C Jaeger, Gregory Wozniak, Emily B Levitan , Lisandro D Colantonio. JAMA 2020 Sep 22;324(12)
Suggest to review that statement.

**Response** : We have revised this statement and cited the two reference in the introduction section.

**Reviewer #2:**

This is a well researched article that needs minor revision to make it more useful and easier reading for readers.

1. All abbreviations in the Abstract, Text and Legends should be preceded by full spelling. To make it easier for readers, a list of abbreviations should be added before the Introduction of the main text.

**Response 1**: We have added full spelling about the abbreviations in the Abstract, Text and Legends. A list of abbreviations was added before the Introduction of the main text.

2. In calculating the average annual percent change (AAPC), the author assumes that the trend is consistent over the period studied, with a statistical calculation used to give the average rate. However as can be seen from Figure 1, there is no consistent trend over the period studied. For example, for newly diagnosed hypertension, the incidence dropped from 1999-2014, and then rose from 2014-2018. Authors therefore need to rewrite the section on "Trends in the prevalence of awareness, treatment, and control among hypertension", to apply AAPC selectively over specific periods or not to use it for certain parameters. It is very important not to over, under or incorrectly estimate consistencies in the trend.

**Response 2**: We used annual percent change (APC) to evaluate the internal trend of each independent interval before and after inflection (Line 168-180), and rewrite the section on "Trends in the prevalence of awareness, treatment, and control among hypertension"(Line 211-213, 235-241, 254-261).

3. Figure 1 is most important, and graphically represents what the whole article is about. It should be presented as 3 separate Figures.

**Response 3**: We have separated into 3 Figures.
